# The Use of Linked Administrative Data to Examine the Impact of Adverse Childhood Experiences: A Scoping Review of the Literature

**DOI:** 10.1177/15248380251383934

**Published:** 2025-11-19

**Authors:** Jacqueline Gannon, Ian Li, Charley Budgeon, Megan Bell

**Affiliations:** 1 University of Western Australia, Perth, Australia; 2 Curtin University, Perth, WA, Australia

**Keywords:** linked administrative data, adverse childhood experiences, life-course outcomes

## Abstract

This review explored the use of linked administrative data to study outcomes associated with adverse childhood experiences (ACEs), which are traumatic events during childhood. A search of Medline, PsycINFO, Embase, ERIC, Education Source, and CINAHL Plus identified studies published from 1998 to 2023. To be included, studies had to use linked administrative data to determine ACE exposure in individuals aged 0 to 18 years, focusing on outcomes categorized as Health, Education, Welfare, or Justice. Following deduplication, title and abstract screening, and a full text review, a total of 309 studies met the inclusion criteria. The review summarized study characteristics such as publication year, geographic location, sample size, age of participants, type of ACE exposure, and outcome studied. An increasing trend in ACE-related publications over the review period was evident, with sample sizes most commonly ranging from 5,001 to 50,000 individuals. The majority of studies utilized data sourced in Australia and the USA, and the participants were predominantly aged 0 to 2 years, with fewer studies involving older children. Over half of the included studies focused on single ACE exposures, particularly sexual abuse, either as a standalone factor or part of broader child maltreatment reports. ACE exposure was most often identified at the level of the “parent/carer,” with mothers more likely to be included when a single parent only was analyzed. Health outcomes were the most frequently studied. While linked administrative data provides a powerful tool for examining multiple exposures and outcomes, the review found a potential under-utilization of its full potential, with fewer studies addressing multiple exposures and outcomes simultaneously.

Adverse childhood experiences (ACEs) is an umbrella term for preventable, potentially traumatic events that occur in childhood from the ages of 0 to 17 years (National Center for Injury Prevention and Control, 2021), and has been the subject of research since the late 1990s. In the seminal ACEs study, Felitti et al. (1998) surveyed adult participants to retrospectively determine their exposure to particular traumatic events in childhood including psychological, physical, or sexual abuse, as well as exposure to various categories of household dysfunction (parental substance abuse, parental mental illness, mother treated violently, or parental criminal behavior). The questionnaire used in the Felitti study has been the foundation for subsequent research (Craig et al., 2023; Matsuura et al., 2013; Reuben et al., 2016) and the original categories have since been expanded to include other ACEs (Ford et al., 2016; Loxton et al., 2019). The additional proposed ACEs have been summarized by Karatekin and Hill (2018), Sasaki et al. (2024), and SmithBattle et al. (2022). They include such exposures as community dysfunction, perceived discrimination, bullying, parent separation or divorce, parent death, and economic hardship; however, there is still disagreement about what should be included in the expanded list of ACEs.

A large body of evidence links ACEs with poor life-course outcomes including reduced education and employment prospects (Dworsky & Courtney, 2001), poor mental (Bager et al., 2020), and physical health (Toivonen et al., 2020), and adverse outcomes such as homelessness and criminal justice involvement (Malvaso et al., 2022). Data on ACEs, however, are most often collected through self-report methods, which can be subject to limitations. Use of population-level administrative data can overcome some of these limitations, potentially providing a more comprehensive and objective understanding of the risks and outcomes associated with ACEs.

This scoping review aims to synthesize research that uses linked administrative data to study ACEs, identify gaps in the existing evidence, and inform data-driven strategies for prevention and intervention.

## How Data Have Been Used to Identify ACEs

A large and growing body of literature on ACEs exists (Jaen et al., 2023; Nikulina et al., 2021; Walsh et al., 2020), but the evidence base is subject to several challenges. Firstly, published studies predominantly use self-report data to measure ACEs, which can be problematic due to potential social desirability bias, the participant’s mental state at the time of being questioned (Colman et al., 2016; Hardt & Rutter, 2004; Lacey et al., 2020; Negriff et al., 2017), or the impact of biasing personality factors on what is reported (Reuben et al., 2016). In their meta-analysis, Francis et al. (2023) examined whether there was overlap in subjective or objective measures of childhood adversity. They found that subjective experiences of individuals only moderately correlate with objective recordings of adverse childhood events. Furthermore, individuals who self-reported childhood adversity were more likely to self-report psychopathology than individuals who only had objective records of childhood adversity, suggesting potential recall, reporting, or shared method bias. In addition, research comparing retrospective and prospective information about ACEs found that adults with psychiatric conditions and those with self-reported health problems are more likely to recall negative experiences in childhood (Reuben et al., 2016). This finding is supported by other studies (Breton et al., 2022; Colman et al., 2016; Hardt & Rutter, 2004). Reuben et al. (2016) report that the reverse can also be true: adults who perceive themselves as free from difficulties—whether physical, mental, cognitive, or social—tend not to acknowledge their early life challenges, even when historical records indicate childhood adversity (Reuben et al., 2016). Furthermore, the time between adult study participants being questioned and the events’ occurrence can result in recall bias and an over or underestimation of the ACE exposure (Negriff et al., 2017). Evaluation of ACEs using more objective methods is therefore needed.

The comparatively recent emergence of linked administrative data as a source for researchers presents an innovative and objective way to determine exposure to ACEs. Administrative data can be described as information that has been collected for registration, transaction, and record keeping (Woollard, 2014), sometimes referred to as a “by-product of government services” (ADR UK, 2023), and includes data that are collected as part of ongoing operations (Statistics Canada, 2023). There are some limitations to the use of linked administrative data for research, such as incomplete capture of sub-populations due to confounding factors (e.g., non-engagement with services, under-reporting), attrition due to migration and/or death, clerical errors, over-representation of certain demographic groups (e.g., Indigenous people) due to systemic bias or racism, omission of key variables of interest, and lengthy approval processes (Christen & Schnell, 2023; Dougall et al., 2020; Harron et al., 2017). Despite this, however, there are many advantages. Firstly, traditional survey-based data collection methods can be cost- and time-intensive, whereas linked administrative data are collected during routine operations, meaning a comparatively reduced cost and a decreased burden on study participants. Administrative data often include entire populations or whole countries (Connelly et al., 2016; Mc Grath-Lone et al., 2022), providing representative and comparable study populations. An additional advantage of administrative data is that different information on the same individuals can be brought together using data linkage methods that protect individual privacy (Jutte et al., 2011; Young & Flack, 2018), allowing the investigation of sensitive topics and inclusion of individuals who may not otherwise participate in research. The potential for study attrition is also minimized, and there is a reduced risk of reporting or recall bias as the data are collected prospectively (Flack et al., 2019; Pattaro et al., 2020). Research using linked administrative data can identify risk or preventative indicators, which could be assessed contemporaneously for data-driven and evidence-based changes to policy and practice. Finally, longitudinally linked administrative data provide researchers with the ability to examine issues with longer-term life-course outcomes or those that are essentially longitudinal, such as social disadvantage or poverty (Pattaro et al., 2020). As governments in many countries routinely collect information on child maltreatment, criminal prosecutions, and physical and mental health diagnoses, there is a significant opportunity to utilize administrative data for the measurement of ACEs.

## The Current Study

This scoping review was undertaken to determine the extent to which ACEs have been examined using linked administrative data. While the authors acknowledge that much of the existing ACEs research has used self-report instruments (Bethell et al., 2017), this review focuses on research using administrative data to measure equivalent ACE constructs. It also focuses on the original ACE categories, as there is still some debate about exactly which childhood adversities should be included in the expanded list.

To the best of our knowledge, this is the first scoping review examining research into the use of linked administrative data to examine ACE exposures on outcomes of Health, Education, Justice and Welfare. It contributes new insights to the current body of knowledge by examining studies where linked administrative data has been the source by which to examine ACEs. Given the expressed issues with the possibility for unreliability with self-reported data, it also investigates the use of routinely collected data as a potentially contemporaneous, comprehensive, and objective measure of ACEs.

The objective of this scoping review was to determine the extent to which linked administrative data have been utilized in research to measure the original ACE categories: psychological, physical or sexual abuse, living with someone with substance use or mental illness issues, having a mother who was treated violently, and having someone in the household exhibiting criminal behavior. Our research questions were as follows.

For publications examining ACE exposure that have used linked administrative data:

1. What are their characteristics (publication year, sample size, country of origin)?
2. What are the ages of ACE exposure that have been most commonly examined?
3. Which ACE exposures have been operationalized?
4. Which outcomes have been investigated?
5. Are there outcomes associated with particular ACEs?

## Methods

### Eligibility Criteria

As the original ACE study was published in Felitti et al. (1998), searches were restricted to publication dates from 1998 to 2023. The scope was also limited to English language publications. Publication types such as comments on other articles, conference proceedings, conference/forum abstracts, meta-analyses, systematic, scoping or literature reviews, incomplete studies, research proposals, study protocols, and dissertations were not included. Studies included in the final scoping review had human participants who were children/adolescents (aged 0–18 years) at the time of exposure to the ACE. Studies reporting research on an ACE exposure not included in the original Felitti study were also excluded. ACE exposure had to have been determined through administrative data; however, there was no such restriction on the measurement of outcomes. There was also no restriction on the country of the research data source, nor the administrative data agency. For inclusion in the final scoping review, studies had to include one of the original ACEs as either a primary exposure or covariate with associated estimates reported; studies where the ACE was “adjusted for,” without reported estimates were excluded. Papers measuring the prevalence of ACEs without reporting an association with an outcome were excluded.

### Information Sources and Search Strategy

Prior to conducting the literature search, several consultation sessions with a librarian from the University of Western Australia were undertaken (in January and February 2024). These sessions developed and informed the search strategy and the selection of databases, clarified and determined search terms, and finalized inclusion and exclusion criteria.

The choice of databases searched for this scoping review was informed by several papers that focused on ACEs research (Donagh et al., 2022; Karatekin et al., 2022; SmithBattle et al., 2022; Tzouvara et al., 2023) and confirmed with the librarian. Databases searched for this scoping review included Medline, PsycINFO, Embase, ERIC, Education Source, and CINAHL Plus.

In response to the scoping review objectives, search terms were selected to capture research on ACEs using linked administrative data. Study search terms and inclusion and exclusion criteria were informed by the work of previously published scoping and systematic reviews on the subject of ACEs (Deighton et al., 2018; Ford et al., 2019; Hinnen et al., 2024; Karatekin et al., 2022; SmithBattle et al., 2022; Tzouvara et al., 2023; Zhang et al., 2023). To determine search terms on the topic of linked administrative data, scoping reviews related to this subject were also reviewed (Allnatt et al., 2022; Han et al., 2023; Lut et al., 2022; Meurk et al., 2021; Yamana et al., 2023) together with a paper on population data (Christen & Schnell, 2023). Supplemental Table 1 provides a complete list of the specific terms used in the final scoping review database search.

This scoping review utilized Covidence Systematic Review Software (2023). Database searches were conducted on February 4 and 11, 2024 undertaken by the first author (JG) and the results were imported into Covidence. The review process occurred from February to October 2024. If papers returned from the database searches were dissertations, conference papers, abstracts or protocols for future studies, a further search was manually undertaken to source published papers resulting from the work and authors. In many cases, resulting papers had already been included through the initial searches and bulk import. However, where this had not occurred, the papers were manually imported.

### Selection of Sources of Evidence

All authors had access to the Covidence online software used for this review. Papers were reviewed, initially at the Title and Abstract Screening stage, then, with the remaining papers, through a Full Text Review to determine inclusion or exclusion. Papers were primarily reviewed by the first author, with clarification sought as required from the co-authors. Agreement on the inclusion or exclusion of these papers was universal.

Critical information was extracted during the full text review and recorded in the final scoping review table (Supplemental Table 2). JG was responsible for the extraction and charting of key variables from included studies. Characteristics reported in tabular form include publication year, paper title, sample size, geographic location (by country and region or state if available), age of ACE exposure, details of the exposure (including categorization by original ACE type), and the outcome (categorized under the headings Welfare, Justice, Health, Education).

### Data Items

Reviewed papers were categorized using the original ACEs categories. Research into issues of parental alcohol or drug misuse were categorized as ACE *Substance Abuse*. Studies investigating parental psychiatric morbidity, mental disorders or illness, post-traumatic stress disorders, suicide attempts, or mothers taking selective serotonin reuptake inhibitors during pregnancy were categorized as ACE *Mental Illness*. For this review, the ACE category of *Domestic Violence* (termed as “Mother Treated Violently” in the Felitti et al., 1998 paper) included papers with exposures of family and domestic violence and assaults on mothers during pregnancy. The ACE *Criminal Behavior* included research on parents who were incarcerated, or who had a history of involvement with police or criminal offending,

Where the ACE exposures of Psychological Abuse, Physical Abuse, and Sexual Abuse were specified as the subject of research in isolation, papers were categorized as such. Although not specifically captured in the original ACEs, this review also included research on children in out-of-home care. This is because evidence from the United States and Australia indicates that the majority of children in care have also experienced child maltreatment (Drake et al., 2022; Harris et al., 2024) and have often experienced longer periods of higher intensity maltreatment from earlier ages than children who have experienced maltreatment but not been placed in care (Harris et al., 2024). For this scoping review, therefore, research examining child maltreatment, child protection involvement, “looked after” children—a term used in the United Kingdom to describe children in the care of their local authority (Fleming et al., 2021)—or children in foster care/out-of-home care, without disaggregating the specific type of abuse, was recorded as ACE *Physical*, *Psychological*, *Sexual Abuse*.

## Results

### Study Selection

Figure 1 presents the PRISMA diagram. Database searches yielded a total of 6,638 papers, which were imported for screening. Covidence removed 3,223 papers through its automated deduplication process, and a further 7 papers were identified as duplicates and manually removed by JG, leaving 3,408 studies for review. An initial screening of titles and abstracts of all 3,408 papers was conducted to exclude any items that did not meet the inclusion criteria. Approximately 40% of papers initially imported (*n* = 2,667) were excluded at this stage as it was possible to determine, from the title and abstract only, that they did not meet the scoping review inclusion criteria. Some studies, for example, did not contain any childhood exposures, and others did not include the original ACEs as exposures. Subsequently, through full-text appraisal of the remaining 741 papers, inclusion or exclusion was determined, with 118 studies excluded as the ACE exposure was self-reported and a further 81 studies excluded as the exposure was not an original ACE. A total of 309 studies were deemed eligible for the scoping review.

**Figure 1.**
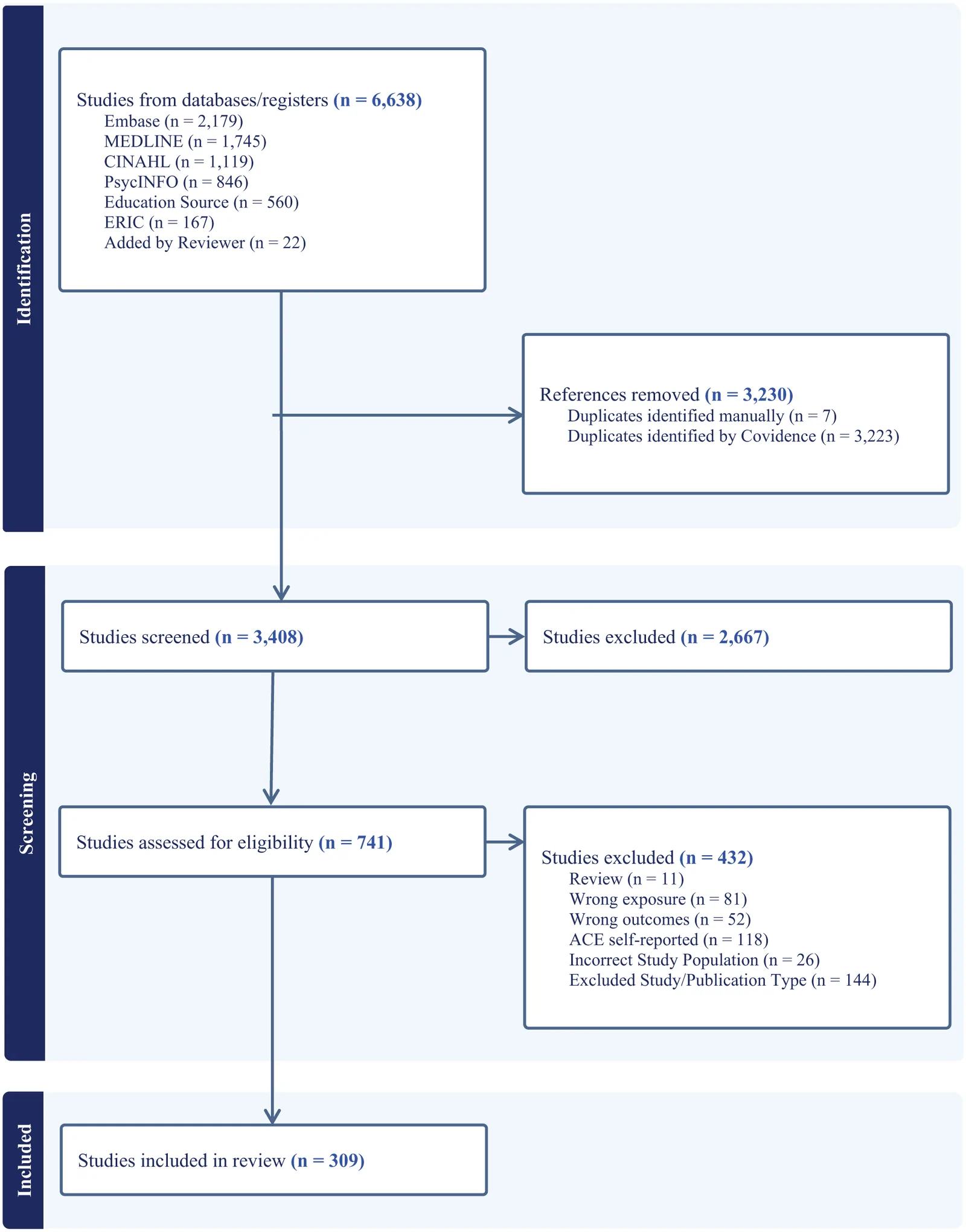
Prisma flow diagram.

### Characteristics of Included Studies

The full scoping review table (Supplemental Table 2) presents the characteristics of the included studies in detail. Table 1 provides a summary of the included studies by publication year, study population size and country of data origin. The number of published papers (by year) examining ACEs using linked administrative data has increased from the time of Felitti’s original paper, with the highest number being published in 2023. The majority of studies were conducted on sample sizes between 5,001 and 50,000 individuals, with one paper not stating the study population sample size. The smallest sample was *N* = 75 (Douglas et al., 2016) and the largest was *N* = 27,568,056 (Brown, 2020). Most utilized data from Australian (*n* = 123, 40%) or U.S. (*n* = 101, 33%) administrative sources. Studies with data sourced from Canada (*n* = 22) and Denmark (*n* = 21) had a similar number of papers in the final scoping review, followed by Sweden (*n* = 17) and the United Kingdom (*n* = 11). Hong Kong, Japan, The Netherlands, Türkiye, and Iceland each had one paper included in the review.

**Table 1.** Summary of Included Papers.

Publication Year	*n*		Size of study population	*n*	Country of origin	*n*
1998		1	0–100	5	Australia	123
1999		1	101–500	25	USA	101
2000		2	501–1,000	15	Canada	21
2001		3	1,001–5,000	56	Denmark	21
2002		1	5,001–50,000	98	Sweden	17
2003		1	50,001–100,000	44	United Kingdom	11
2004		1	100,001–500,000	38	Finland	4
2005		1	500,001–1,000,000	15	New Zealand	3
2006		3	>1,000,000	12	Israel	2
2007		3	Not specified	1	Taiwan	2
2008		4			Hong Kong	1
2009		3			Japan	1
2010		12			The Netherlands	1
2011		8			Türkiye	1
2012		12			Iceland	1
2013		12				
2014		9				
2015		13				
2016		18				
2017		29				
2018		27				
2019		28				
2020		28				
2021		22				
2022		28				
2023^a^		39				

a 2023 includes four papers accepted in 2023 but published in 2024.

#### Age of ACE Exposure

Figure 2 summarizes the age of ACE exposure for the included studies. Ages are disaggregated as pregnancy/prenatal, birth-infancy (0–2 years), early childhood (2–5 years), middle-late childhood (5–12 years), and youth-teenager (12–18 years) or child/childhood if no specific age was stated. The details for individual studies can be found in Supplemental Table 2. Some studies listed specific ages of ACE exposure. Hash et al. (2020), for instance, investigated maltreatment exposures of children aged 10 to 24 months. This study was coded as occurring in the range of 0 to 2 years. Other research considered exposure ages, which spanned several categories. Where this occurred, the exposure was coded for each of the covered age ranges. One example is the work of Fleming et al. (2021), which used exposure ages of 4 to 18 years. This study was coded as having participants in the ranges of 2 to 5, 5 to 12, and 12 to 18 years. Where the exposure period was listed as perinatal, the study was scored as both prenatal and birth-infancy (0–2 years; e.g., Melis et al., 2023; O’Donnell et al., 2015). Rather than specify a specific age range, 42 studies referred to the exposure time as “child” or “childhood” (e.g., the work of Hasson et al., 2022). These papers were coded as Child/Childhood. A similar approach was utilized when studies referenced exposure periods for participants as infancy, early childhood, middle childhood or the teenage years rather than specifying age ranges (Andersen & Wildeman, 2014; Karriker-Jaffe et al., 2018; Smith et al., 2024; Whitten et al., 2019). In the final scoping review, the age range with the largest number of studies included some reference to ACE exposure between the ages of 0 and 2 years (*n* = 202, 25%), with fewer papers for older children (early childhood *n* = 178, middle-late childhood *n* = 166, youth-teenager *n* = 139).

**Figure 2.**
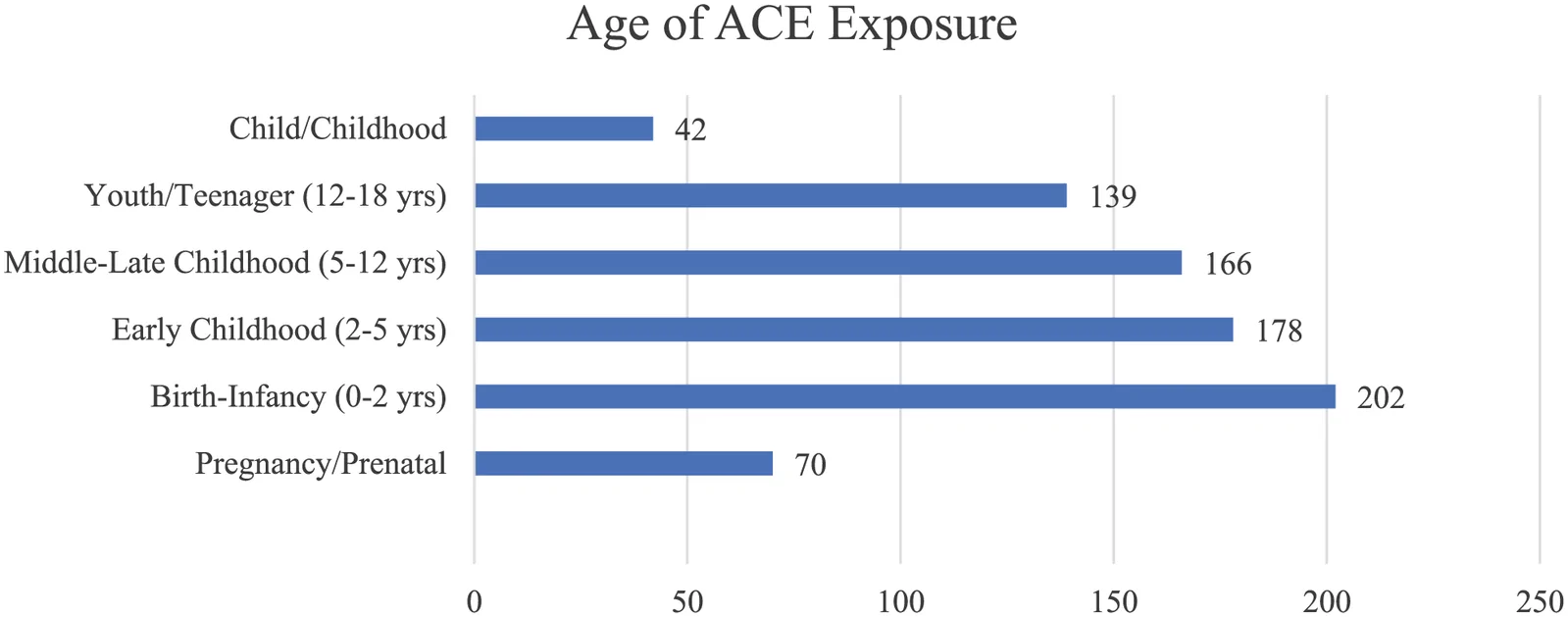
Age of ACE exposure. *Note.* ACE = adverse childhood experience.

A number of studies were focused on the specific periods where exposures were most impactful. In their study, for example, Stewart et al. (2008) examined the impact of the timing and chronicity of childhood maltreatment on youth offending. For this study, the timing of exposure was categorized as early childhood only (birth–5 years), late childhood only (6–11 years), adolescent only (12–17 years) or persistent maltreatment, where the ACE was evident in both childhood and adolescence. A similar approach was used in research on youth offending undertaken in the Northern Territory, Australia (He et al., 2021), where the timing of maltreatment or out-of-home care exposure was categorized as 0 to 4 years, 5 to 9 years, or both.

#### Type of ACE

The disaggregation of included studies by ACE exposure is presented in Figure 3. The most common ACE exposure category was sexual abuse (*n* = 162, 20%). Domestic violence was the ACE exposure that was the least frequently examined (*n* = 34, 4%).

**Figure 3.**
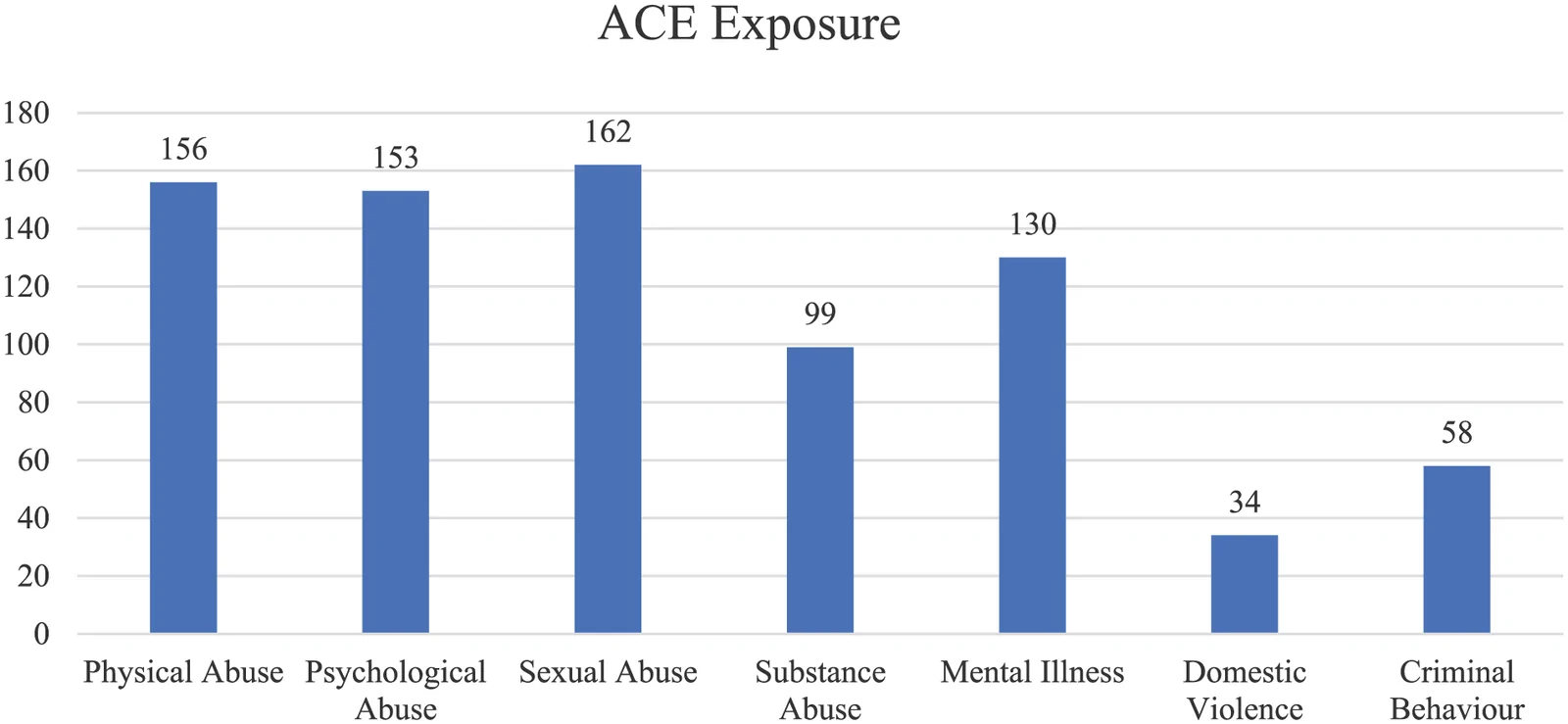
Papers by ACE exposure. *Note.* ACE = adverse childhood experience.

The ACE category of household dysfunction measures ACEs at the level of the parent (i.e., parent mental illness, substance abuse, criminal behavior, and domestic violence), but considers the impact of these exposures on the child. The breakdown of these can be seen in Table 2. Most of the studies included in the scoping review did not identify mothers and fathers separately, instead referring to “parent” or “carer.” In papers with an ACE exposure of Mental Illness, for example, 73 (56%) of the included studies investigated whether a child had a parent or carer with such exposures as a psychiatric disorder (Ranning et al., 2022), a mood disorder (Cameranesi et al., 2022), or psychiatric illness (Elsenburg et al., 2023). Where papers included models for both the mother and father in the one study, the paper was categorized as Parent/Carer (for ACE exposure). For example, in the work of Athanassiou et al. (2023) and O’Hare et al. (2022), the researchers examined the impact of maternal or paternal criminal behavior, and maternal or paternal mental illness. If a specific parent was identified through linked administrative data, it was more frequently the mother than the father. For example, maternal mental Illness was the focus of 53 studies (91%), whereas paternal mental Illness was only examined in 5 studies (9%).

**Table 2.** Papers by ACE Exposure (Adult).

Parent-level exposure	Parent/carer	Father	Mother
Mental illness	73	5	53
Substance abuse	57	0	28
Criminal behavior	44	2	11
Domestic violence	31	0	7

*Note.* ACE = adverse childhood experience.

#### Number of ACEs

Figure 4 compares the included studies by number of examined ACE exposures—one versus more than one. The majority (*n* = 203, 66%) of the 309 included studies considered only one exposure, and this was most often child welfare (physical abuse, psychological abuse, sexual abuse; *n* = 107, 52%). The remaining 106 papers considered the impact of 2 or more ACE exposures.

**Figure 4.**
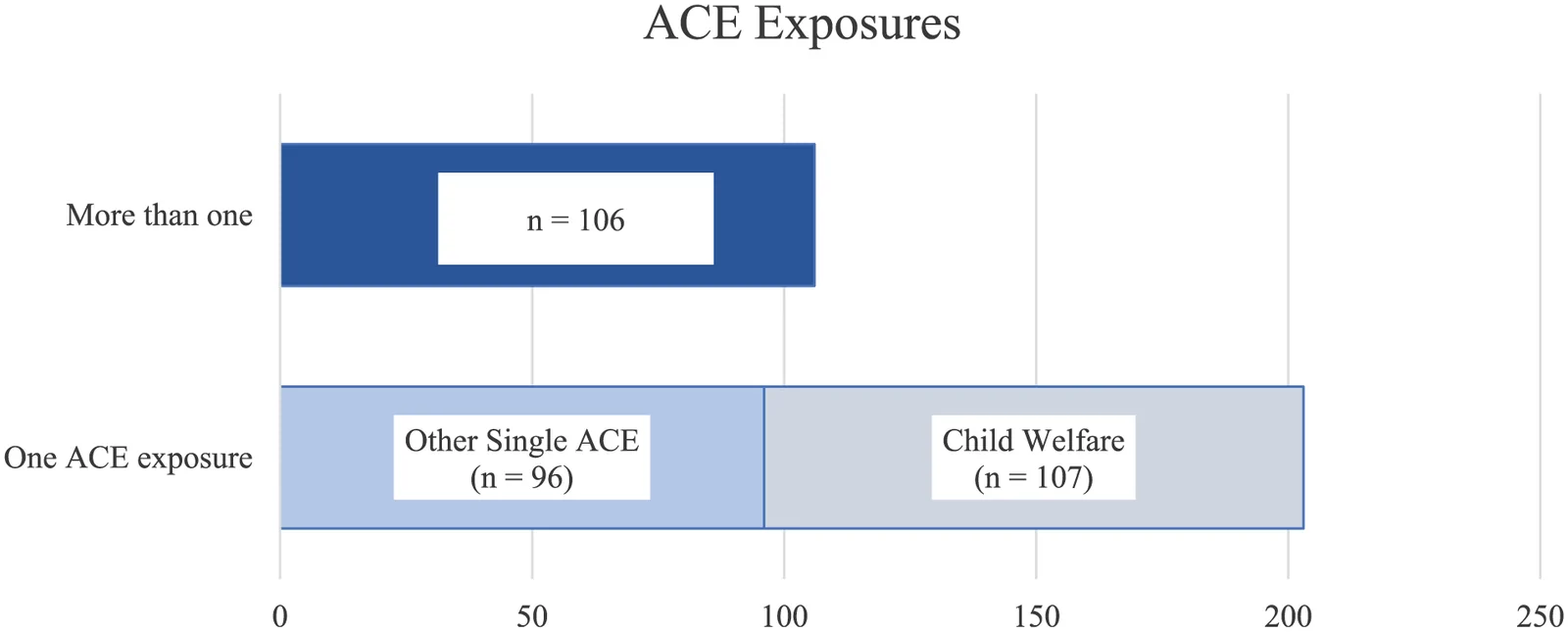
ACE exposure—one versus two or more. *Note.* ACE = adverse childhood experience.

#### Outcomes Associated with ACEs

We were also interested in identifying the outcomes for children exposed to ACEs. The counts of papers examining the impact of ACEs on the outcomes of Health, Education, Justice, and Welfare are displayed in Figure 5. The outcome of Health was the most frequently studied (*n* = 190, 51%), with papers presenting research on the outcome of Justice (*n* = 43, 11%) being the least frequently studied. All included studies found that ACEs were associated with negative life-course outcomes. Negative health outcomes related to ACEs included hospitalization for reasons such as suicide attempt (Ringbäck Weitoft et al., 2008), child mortality (Miyamoto et al., 2017), infectious and respiratory disorders, injury or poisoning (Fitzpatrick et al., 2021; Orr et al., 2020), mental health conditions (Gnanamanickam et al., 2020; Leckning et al., 2023), premature mortality (Di Prinzio et al., 2020), and other physical and psychological morbidities (Toivonen et al., 2020). Examples of Education outcomes considered include academic results (Bell et al., 2023b), school suspensions (Bell et al., 2021) and attendance (Orr et al., 2023), and school non-completion (Jay et al., 2022). For the outcome of Justice, studies examined criminal offending, including juvenile delinquency and other contacts with the justice system (Lee & Jonson-Reid, 2009; Valuri et al., 2017; Yampolskaya et al., 2011), and anti-social behavior (Jonson-Reid et al., 2010).

**Figure 5.**
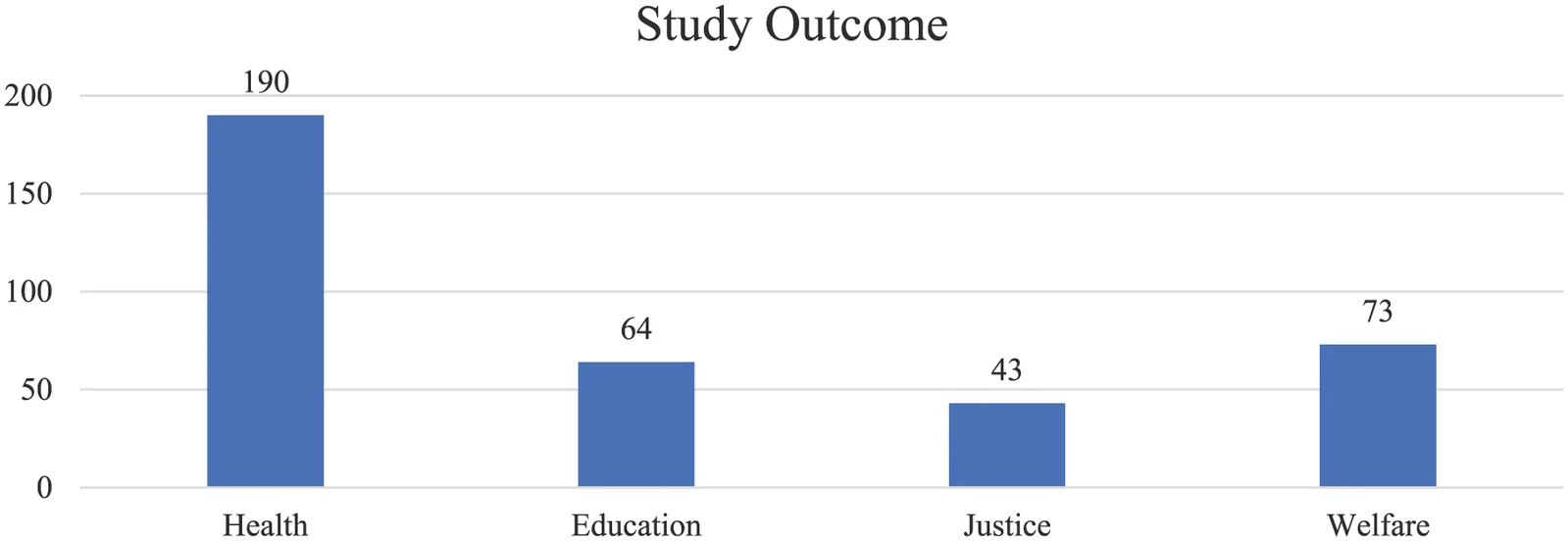
Papers by study outcome.

Most of the included studies were focused on only one category of outcome (*n* = 261, 84%; Figure 6), but a number of studies examined the impact of particular ACEs on multiple outcomes. For example, one study determined that exposure to a maternal alcohol-related diagnosis increased the odds of three or more negative outcomes across Health, Education, Justice, and Welfare (O’Leary et al., 2020). Similarly, Brännström et al. (2020) determined that children in long term foster care before the age of 13 years had an increased likelihood of poor outcomes in Education, Health, and Justice. Two examined all four outcomes (Health, Education, Justice, and Welfare). One study (Malvaso et al., 2022) modeled outcomes for public housing, hospitalization, and emergency department presentations, education, and justice contacts. The other (O’Leary et al., 2020) modeled neonatal health, school failure and poor school attendance, justice contacts, and child protection involvement.

**Figure 6.**
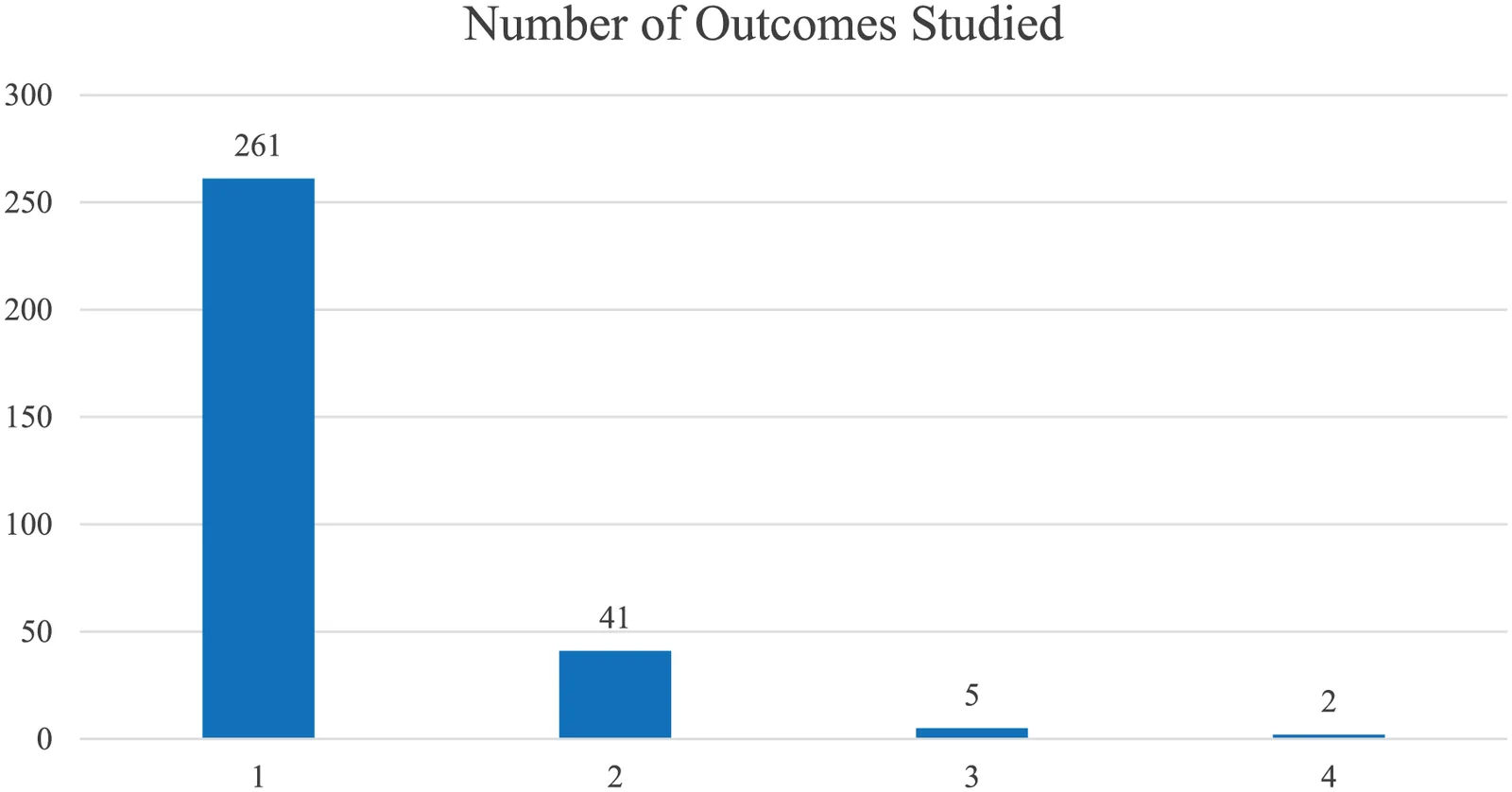
Papers by number of outcomes studied.

To determine if there were particular outcomes associated with different ACEs, we analyzed the included studies’ outcomes by ACE exposures (Table 3). Where studies had multiple exposures, and/or multiple outcomes, they were included in each of the appropriate categories. In addition to being the most studied outcome overall (see Figure 6), Health was also the most frequently investigated outcome across all ACE exposure categories. The outcomes investigated in association with the ACE combined category Physical, Psychological, Sexual Abuse were predominantly related to Health (*n* = 80, 44%). One example, the research of Font et al. (2019), examined the risk of teenage motherhood for children who had been in foster care. Similarly, the outcomes investigated in association with the ACE Criminal Behavior were primarily Health outcomes (*n* = 33, 46%). An included paper in this category examined neonatal abstinence syndrome and other neonatal outcomes for babies born to incarcerated or formerly incarcerated women (Bell et al., 2023a). Research examining the ACE Mental Illness also primarily investigated Health outcomes (*n* = 85, 56%). For example, Nilsson et al. (2008) examined the association between maternal mental illness and infant death. The outcome most studied through the ACE exposure of Domestic Violence was Health (*n* = 17, 41%), which was comparable with the number of papers from the outcome category of Welfare (*n* = 15, 36%). Here, the work of Orr et al. (2019) contributes insights on the increased risk of child protection involvement when mothers have been hospitalized for assault. Papers with an ACE exposure of Substance Abuse also primarily investigated Health outcomes. As one illustration of this category, the work of McCutcheon et al. (2019) found that the risk of infant and child mortality is increased for babies born to mothers who have multiple drink driving offenses.

**Table 3.** Outcomes by ACE Exposures Measured.

ACE	Outcome			
	Health (*n*)	Education (*n*)	Justice (*n*)	Welfare (*n*)
Physical, psychological, sexual abuse (combined)	80	36	28	37
Sexual^a^	7	0	5	1
Physical^a^	2	1	1	0
Psychological^a^	0	1	0	0
Parent criminal behavior	33	16	5	17
Parent mental illness	85	30	8	29
Parent domestic violence	17	5	4	15
Parent substance abuse	52	17	7	36

*Note.* ACE = adverse childhood experience.

a Single exposure only.

## Discussion

This scoping review aimed to summarize research using linked administrative data to measure ACEs and their associated outcomes. To the best of our knowledge, this paper is the first scoping review on this topic, providing insights into the potential for further utilization of linked administrative data in ACEs research.

This scoping review examined studies by publication year, sample size, and country of origin for the data. We found that the number of included studies generally increased in the years from 1998 to 2023. There are several plausible explanations for this trend. The Australian Institute of Health and Welfare reports that the last decade has seen a number of advancements in the broad data landscape. These include a move from traditional to electronic recording methods, technological advancements supporting the ability to link records and acknowledgment that data must be appropriately governed and safely managed (Australian Institute of Health and Welfare, 2024). Additionally, new datasets are constantly being added to existing linked data systems across the world, including historical records, and records that are being newly digitized. Although administrative data are not generally collected or intended for research purposes, there appears to be a developing inclination toward the acceptance of their potential in research. In the United States, for example, the National Bureau of Economic Research provides readily downloadable files for some of their datasets (National Bureau of Economic Research, 2024), and in Australia, the Person Level Integrated Data Asset (PLIDA) provides data from securely combined information on health, education, government payments, income and taxation, employment, and population demographics for researchers (Australian Bureau of Statistics, n.d.). It is possible that the increased trend in the annual number of papers using linked administrative data to research ACEs is a result of improvements in the range of available data and advances in access for researchers.

Studies with between 5,001, and 50,000 participants represented the largest group of included papers in this scoping review. Though population sizes vary, many administrative datasets with linkage potential or those that are already linked are substantial. For example, data for a single year cohort of children enrolled in preschool programs in Australia results in a sample size of 337,305 (Australian Bureau of Statistics, 2024), demonstrating significant scope to examine exposures in large population samples. Such large sample sizes with whole of population data also present an opportunity to identify rare exposures or outcomes that may be missed with smaller cohorts. A further benefit of using whole of population administrative data is that representativeness is not problematic, as, by definition, findings are generalizable to the population.

An additional frame of reference gained from our scoping review was the consideration of the country of origin for the data used. The final selection of papers predominantly included studies undertaken in Australia and the USA. In their paper on data linkage for evidence-informed policy, Panteli et al. (2023) selected Australia and the USA as exemplar case studies for their approaches to data availability and access. The large number of scoping review papers from these countries may reflect a commitment to link and utilize administrative data for research purposes. It is also possible that countries with lower incomes lack the established infrastructure and resources required for the collection of large-scale data. The World Bank provide information on the performance of statistical systems across the globe (World Bank Group, 2025). Their framework includes measures for data use, services, products, sources and infrastructure. In this ranking, the majority of African nations, and countries such as Nicaragua, Venezuela, Yemen, and Papua New Guinea are ranked in the bottom 20%. The authors acknowledge that the findings of this scoping review highlight the need for improved data systems in these countries to ensure a globally comprehensive representation of the impact of ACEs.

Our review also explored ACE exposures and the outcomes associated with them. We categorized the included studies by age of ACE exposure. The greatest number of papers included in this scoping review examined exposures in young children (aged 0–2 years). This finding may be due to the accepted principle that the early childhood period is a critical time for brain and cognitive development, language acquisition, social and emotional learning, and increased complexity in motor skills (Black et al., 2017). This period of rapid brain development could be adversely impacted by negative experiences, such as ACEs, and these impacts can be long lasting (Rahapsari & Levita, 2024; Regmi et al., 2023). It is plausible that many researchers, who are passionate about mitigating the vulnerability associated with ACEs, choose to focus their investigations on stages of childhood where there is the greatest prospect of improving outcomes. Our finding that the number of included studies reduced as the exposure age increased may suggest an opportunity for further research in this area. Depending on the available data, it may be possible to examine multiple age range categories to determine the point in a child’s development where the risk of vulnerability to certain outcomes is higher.

In addition to the age of ACE exposure, we analyzed the ACEs which were most frequently studied. We found that Child Welfare, particularly sexual abuse, was the most frequently examined ACE. This finding could be attributed to mandatory obligations for reporting of child abuse, resulting in good data capture for this exposure. It may also be due to the acknowledged significant and long-lasting impact of child abuse, making this ACE exposure the focus of more studies. On the other hand, the comparatively infrequent examination of parent-level ACEs using linked administrative data could be due to non-availability of familial linkage in some countries, impeding the ability to examine outcomes associated with parent-level ACE exposures. In research that did examine parent-level exposures using linked administrative data, the largest proportion of studies considered the child’s parent/carer, without specifying the sex of the parent. Where a single parent was identified, it was more likely to be the mother. This disproportionality is likely due to a limitation of linked administrative data, where a mother’s details are always recorded on the birth record, but paternal details may be missing, preventing the linking of father and child records. Research has established that children with missing paternal information on the birth record are at high risk of adverse outcomes (Sims & O’Donnell, 2015); omission of fathers from research on ACEs could therefore bias the findings. In their scoping review examining the methods to link fathers in administrative data, Lut et al. (2022) found that Denmark, Sweden, Finland, and Norway lead the world in identifying fathers through data linkage. Our scoping review suggests that more work is needed to link fathers in administrative data to more comprehensively evaluate parent-level ACE exposures and associated child outcomes.

The linkage of administrative data typically enables the inclusion of data from multiple agencies or organizations with many possible variables to consider. Most studies in our scoping review, however, considered only one exposure and one outcome. It is possible that researchers approach a study with a narrow field of interest in terms of the exposure they are considering, and it is also plausible that access to a broader data set was unavailable. It is nevertheless problematic to estimate a complex causal relationship when only single exposures are modeled. This is especially relevant considering that evidence of the compounding and clustering nature of multiple adversities is well-established. For example, some parents who are experiencing adversity discuss their own experiences of childhood trauma, and also relate issues of substance use, mental health issues, and abusive or violent relationships (Allen et al., 2024; Kedzior et al., 2024). It is also accepted that many incarcerated children and adults have multifaceted adversity profiles (Henry, 2020; Neagoe & Papasteri, 2023). Consideration of multiple ACE exposures in research is necessary for a comprehensive understanding of the pathways to adverse outcomes.

When examining outcomes related to ACEs, we found that Health was the outcome most frequently investigated, both overall and for each distinct category of ACE exposure. It is possible this is due to health records being well-established as an administrative data source and readily linked to other data sets. There were fewer studies, across all ACE exposures, that examined outcomes for Education, Welfare, and Justice. This potentially represents an under-utilization of existing linked data to determine the full extent of the longer-term impact of ACEs.

We acknowledge that the identified associations between ACEs and poor life-course outcomes are probabilistic and not deterministic. It is true that, with appropriate supports, children who have experienced ACEs can have successful and productive lives, though this is not always captured in research using administrative data. Without exception, however, the evidence from the papers included in this scoping review supports the theory that ACEs negatively impact a variety of life-course outcomes, and similar findings are presented in research where ACEs are self-reported. The use of routinely collected, whole of population, administrative data, however, provides an independent and potentially contemporaneous method to determine the risk of ACEs for individuals. The integration of linked administrative data with self-report data would increase capacity to examine pathways to ACEs and their associated outcomes, as well as protective and resilience factors, in detail (Lut et al., 2022). Finally, linked administrative data provides an opportunity to objectively examine multiple exposures and multiple outcomes without additional cost or burden to participants.

### Limitations and Strengths

While this scoping review contributes new insights, it nevertheless has limitations. Firstly, the search was restricted to studies published in English. It is possible that further research has been undertaken and published in other languages that were not included in this scoping review. The use of Felitti et al.’s (1998) ACEs as the focus for this scoping review may be interpreted as excluding important ACEs that have more recently been added to the list of original exposures; however, given the wide use of the original categories in ACEs research, and the contention regarding what should be included in the expanded categories, the pragmatic decision was made to focus on the original list. A logical progression would be further investigation into the use of linked administrative data to examine a broader range of ACEs. The authors also acknowledge that no data collection method is capable of capturing all instances of adversity. Though there are many benefits to using linked administrative data in ACEs research, incomplete data capture remains an issue, particularly in relation to undisclosed or unreported child abuse, untreated mental illness, or undetected criminal behavior. Over-representation of particular population groups in some administrative datasets due to systemic bias inherent in policies and practices (e.g., increased surveillance of specific ethnic or racial groups resulting in increased child maltreatment reports) is also a challenge requiring further consideration. Further, while a limitless search would have been unmanageable, this scoping review included searches using only six databases. Though the choice of these databases was informed by the literature, we may have inadvertently omitted papers that were not linked to these databases. Finally, gray literature and other sources may have been able to contribute additional insights to our review but were not included in this study. Notwithstanding these limitations, our scoping review offers crucial observations on the existing literature regarding the measurement of ACEs with administrative data, and the evaluation of the association of ACEs with a variety of outcomes. The comprehensive list of search terms, range of included years, and broad capture of both exposures and outcomes contribute detailed and wide-ranging information about the existing literature on this subject.

### Implications of the Scoping Review

This scoping review has identified several potential areas for further research. Given our finding that many studies examining ACEs utilize only one exposure and one outcome, investigations of the interaction of multiple exposure variables and multiple outcomes are important. With increasing access to and expansion of linked administrative data, it would be valuable to conduct further research into some of the areas identified in our scoping review. As two examples, the outcome of Justice was the subject of comparatively fewer studies, as were studies into the impact of ACE exposure at the parent level for fathers. Additionally, it may be beneficial to determine which ACE exposures, both in isolation and in combination, are most impactful and whether there are critical age periods during which different ACEs are associated with specific outcomes. Relatedly, our inclusion of research on out-of-home care in the child welfare category contributed to the high number of papers on this topic in the scoping review. It may be useful, in future studies, to categorize placement in out-of-home care as a separate ACE, and consider the intersectionality of this exposure with other ACEs such as parental incarceration or mental illness. Lastly, although there are many logistical issues to overcome, it would be highly beneficial to develop large scale systems to link self-report data to administrative datasets to best capture the most comprehensive picture of childhood exposures to ACEs and their outcomes.

## Conclusion

This scoping review presents a summary of research using linked administrative data to measure ACEs and investigate the association of ACEs with a range of outcomes across the domains of Health, Education, Welfare, and Justice. Importantly, as many studies considered only one exposure and outcome, our scoping review suggests a possible under-utilization of existing administrative data for research purposes. The comparatively recent emergence of larger linked administrative datasets offers the potential to investigate multiple exposures on multiple outcomes, supporting data-driven, evidence-based, and cost-effective decision making. Additionally, as they are routinely collected, these data provide a mechanism for ACE identification without contributing to response burden in vulnerable populations.

**Table:** 

Scoping review key findings 1. Publication trends, sample sizes, and data origin countries • Studies examining ACEs using linked administrative data have increased since Felitti’s original paper, with the highest number of publications in 2023. • Sample sizes ranged widely, from 75 to over 27 million, with most studies conducted on populations between 5,001 and 50,000 individuals. • Data primarily came from Australian (*n* = 123) and U.S. (*n* = 101) based sources, followed by Canada, Denmark, Sweden, and the United Kingdom. 2. Age of ACE exposure and exposure categories • Studies assessed ACE exposure across various developmental stages (e.g., prenatal, infancy, early childhood, childhood, middle childhood, teenager). • The largest number of studies referenced exposure between ages 0 and 2 years, with decreasing numbers as children aged. • Sexual abuse was the most frequently examined category (*n* = 162), while domestic violence was the least studied (*n* = 34). • Many studies identified parents or carers as the adults responsible for ACEs, with maternal exposures being more frequently studied than paternal. 3. Outcomes of ACEs • Health was the most frequently studied outcome (*n* = 190), followed by Education, Welfare, and Justice. • Some studies explored impacts across multiple domains, revealing associations between ACES and outcomes such as hospitalization, school non-completion, and juvenile delinquency

*Note.* ACE = adverse childhood experience.

**Table:** 

Scoping review implications • Improved data systems and research using linked administrative data to consider the impact of ACEs in countries beyond those identified by our review would ensure findings on this important research topic are more globally representative. • Future research is needed to investigate the interaction of multiple exposure variables and multiple outcomes. • More studies on a wider variety of outcomes would supplement the existing literature, as would exposures at the parent level for fathers. • There would be merit in attempting to determine which ACE exposures, as standalones and also in interaction with other ACEs, increase vulnerability in exposed individuals.

*Note.* ACE = adverse childhood experience.

## Supplemental Material

sj-docx-1-tva-10.1177_15248380251383934 – Supplemental material for The Use of Linked Administrative Data to Examine the Impact of Adverse Childhood Experiences: A Scoping Review of the Literature Supplemental material, sj-docx-1-tva-10.1177_15248380251383934 for The Use of Linked Administrative Data to Examine the Impact of Adverse Childhood Experiences: A Scoping Review of the Literature by Jacqueline Gannon, Ian Li, Charley Budgeon and Megan Bell in Trauma, Violence, & Abuse

sj-docx-2-tva-10.1177_15248380251383934 – Supplemental material for The Use of Linked Administrative Data to Examine the Impact of Adverse Childhood Experiences: A Scoping Review of the Literature Supplemental material, sj-docx-2-tva-10.1177_15248380251383934 for The Use of Linked Administrative Data to Examine the Impact of Adverse Childhood Experiences: A Scoping Review of the Literature by Jacqueline Gannon, Ian Li, Charley Budgeon and Megan Bell in Trauma, Violence, & Abuse

## Data Availability

Exact search terms used for this scoping review have been provided in Supplemental Table 1.

## References

[bibr1-15248380251383934] ADR UK. (2023). What is administrative data? Retrieved 19 November from https://www.adruk.org/our-mission/administrative-data/

[bibr2-15248380251383934] Allen K. Melendez-Torres G. J. Ford T. Bonell C. Berry V. (2024). Parental domestic violence and abuse, mental ill-health, and substance misuse and the impact on child mental health: A secondary data analysis using the UK millennium cohort study. BMC Public Health, 24(1), 2310–2315. 10.1186/s12889-024-19694-1 PMC11346203 39187800

[bibr3-15248380251383934] Allnatt G. Elliott M. Scourfield J. Lee A. Griffiths L. J. (2022). Use of linked administrative children’s social care data for research: A scoping review of existing UK studies. The British Journal of Social Work, 52(7), 3923–3944. 10.1093/bjsw/bcac049

[bibr4-15248380251383934] Andersen S. H. Wildeman C. (2014). The effect of paternal incarceration on children’s risk of foster care placement. Social Forces, 93(1), 269–298. 10.1093/sf/sou027

[bibr5-15248380251383934] Athanassiou U. Green M. Tzoumakis S. Whitten T. Laurens K. R. Harris F. Carr V. J. Dean K. (2023). Relationships between parental mental illness and/or offending and offspring contact with the police in childhood: Findings from a longitudinal record-linkage study. Criminal Behaviour and Mental Health, 33(1), 72–84. 10.1002/cbm.2274 PMC10947495 36683139

[bibr6-15248380251383934] Australian Bureau of Statistics. (2024). Preschool education. ABS. https://www.abs.gov.au/statistics/people/education/preschool-education/latest-release

[bibr7-15248380251383934] Australian Bureau of Statistics. (n.d.). Person Level Integrated Data Asset (PLIDA). Australian Bureau of Statistics. https://www.abs.gov.au/about/data-services/data-integration/integrated-data/person-level-integrated-data-asset-plida

[bibr8-15248380251383934] Australian Institute of Health and Welfare. (2024). Australia’s health data landscape. https://www.aihw.gov.au/reports/australias-health/health-data-landscape

[bibr9-15248380251383934] Bager L. Agerbo E. Skipper N. Høgh Thøgersen M. Laursen T. M. (2020). Risk of psychiatric diagnoses in children and adolescents of parents with torture trauma and war trauma. Acta Psychiatrica Scandinavica, 142(4), 307–318. 10.1111/acps.13203 32564360

[bibr10-15248380251383934] Bell M. F. Glauert R. Ohan J. L. Preen D. B. Bayliss D. M. (2021). Early school suspensions for children with adverse childhood experiences (ACEs). Journal of Applied Developmental Psychology, 76, 101300. 10.1016/j.appdev.2021.101300

[bibr11-15248380251383934] Bell M. F. Kelty E. Segal L. Dennison S. Kinner S. A. Dawe S. Spittal M. J. Preen D. B. (2023a). Neonatal abstinence syndrome and other neonatal outcomes for the infants of women experiencing incarceration: A retrospective cohort study. The Australian Journal of Social Issues, 59(2), 344–357. 10.1002/ajs4.296

[bibr12-15248380251383934] Bell M. F. Segal L. Dennison S. Kinner S. Dawe S. Spittal M. Preen D. B. (2023b). Numeracy and literacy attainment of children exposed to maternal incarceration and other adversities: A linked data study. Journal of School Psychology, 100, 101241–101241. 10.1016/j.jsp.2023.101241 37689438

[bibr13-15248380251383934] Bethell C. D. Carle A. Hudziak J. Gombojav N. Powers K. Wade R. Braveman P. (2017). Methods to assess adverse childhood experiences of children and families: Toward approaches to promote child Well-being in policy and practice. Academic Pediatrics, 17(7), S51–S69. 10.1016/j.acap.2017.04.161 PMC6035880 28865661

[bibr14-15248380251383934] Black M. M. P. Walker S. P. P. Fernald L. C. H. P. Andersen C. T. M. S. DiGirolamo A. M. P. Lu C. P. McCoy D. C. Fink G. Shawar Y. R. Shiffman J. Devercelli A. E. Wodon Q. T. Vargas-Barón E. Grantham-McGregor S. P. (2017). Early childhood development coming of age: Science through the life course. The Lancet (British edition), 389(10064), 77–90. 10.1016/S0140-6736(16)31389-7 PMC5884058 27717614

[bibr15-15248380251383934] Brännström L. Vinnerljung B. Hjern A. (2020). Outcomes in adulthood after long-term foster care: A sibling approach. Child Maltreatment, 25(4), 383–392. 10.1177/1077559519898755 31960707

[bibr16-15248380251383934] Breton E. Kidman R. Behrman J. Mwera J. Kohler H.-P. (2022). Longitudinal consistency of self-reports of adverse childhood experiences among adolescents in a low-income setting. SSM—Population Health, 19, 101205–101205. 10.1016/j.ssmph.2022.101205 PMC9449854 36091299

[bibr17-15248380251383934] Brown R. (2020). The intergenerational impact of terror: Did the 9/11 tragedy Iimpact the initial human capital of the next generation? Demography, 57(4), 1459–1481. 10.1007/s13524-020-00876-6 PMC7442658 32514847

[bibr18-15248380251383934] Cameranesi M. Shooshtari S. Piotrowski C. C. (2022). Investigating adjustment profiles in children exposed to intimate partner violence using a biopsychosocial resilience framework: A Canadian population-based study. Child Abuse & Neglect, 125, 105453–105453. 10.1016/j.chiabu.2021.105453 35032822

[bibr19-15248380251383934] Christen P. Schnell R. (2023). Thirty-three myths and misconceptions about population data: From data capture and processing to linkage. International Journal of Population Data Science, 8(1), 2115–2115. 10.23889/ijpds.v8i1.2115 PMC10454001 37636835

[bibr20-15248380251383934] Colman I. Kingsbury M. Garad Y. Zeng Y. Naicker K. Patten S. Jones P, B. Wild T. C. Thompson A. H. (2016). Consistency in adult reporting of adverse childhood experiences. Psychological Medicine, 46(3), 543–549. 10.1017/S0033291715002032 26511669

[bibr21-15248380251383934] Connelly R. Playford C. J. Gayle V. Dibben C. (2016). The role of administrative data in the big data revolution in social science research. Social Science Research, 59, 1–12. 10.1016/j.ssresearch.2016.04.015 27480367

[bibr22-15248380251383934] *Covidence Systematic Review Software* . (2023). Veritas Health Innovation. https://www.covidence.org/

[bibr23-15248380251383934] Craig F. Servidio R. Calomino M. L. Candreva F. Nardi L. Palermo A. Polito A. Spina M. F. Tenuta F. Costabile A. (2023). Adverse childhood experiences and mental health among students seeking psychological counseling services. International Journal of Environmental Research and Public Health, 20(10), 5906. 10.3390/ijerph20105906 PMC10218713 37239632

[bibr24-15248380251383934] Deighton S. Neville A. Pusch D. Dobson K. (2018). Biomarkers of adverse childhood experiences: A scoping review. Psychiatry Research, 269, 719–732. 10.1016/j.psychres.2018.08.097 30273897

[bibr25-15248380251383934] Di Prinzio P. Abel K. M Ambrosi T. L Jablensky A. Croft M Shah S McNeil T. F Matthias Pierce M Morgan V. A . (2020). Parsing components of risk of premature mortality in the children of mothers with severe mental illness. Schizophrenia Research, 218, 180–187. 10.1016/j.schres.2020.01.006 31980344

[bibr26-15248380251383934] Donagh B. Taylor J. al Mushaikhi M. Bradbury-Jones C. (2022). Sibling experiences of adverse childhood experiences: A scoping review. Trauma, Violence, & Abuse, 24(5), 3513–3527. 10.1177/15248380221134289 PMC10594841 36382953

[bibr27-15248380251383934] Dougall N. Savinc J. Maxwell M. Karatzias T. O’Connor R. C. Williams B. John A. Cheyne H. Fyvie C. Bisson J. I. Hibberd C. Abbott-Smith S. Nolan L. (2020). Childhood adversity, mental health and suicide (CHASE): A methods protocol for a longitudinal case-control linked data study. International Journal of Population Data Science, 5(1), 1338–1338. 10.23889/ijpds.v5i1.1338 PMC7473285 34232970

[bibr28-15248380251383934] Douglas T. Green J. Park J. Turkovic L. Massie J. Shields L. (2016). Psychosocial characteristics and predictors of health-care use in families of young children with cystic fibrosis in Western Australia. Journal of Paediatrics and Child Health, 52(1), 34–39. 10.1111/jpc.13011 26515431

[bibr29-15248380251383934] Drake B. Fluke J. D. Kim H. Orsi R. Stubblefield J. L. (2022). What proportion of foster care children do not have child protective services reports? A preliminary look. Child Maltreatment, 27(4), 596–604. 10.1177/10775595211033855 34308682

[bibr30-15248380251383934] Dworsky A Courtney M. E . (2001). Self-sufficiency of former foster youth in wisconsin: Analysis of unemployment insurance wage data and public assistance data. University of Wisconsin, Madison.

[bibr31-15248380251383934] Elsenburg L. K. Rieckmann A. Bengtsson J. Lange T. Baker J. L. Sørensen T. I. A. Rod N. H. (2023). Early childhood adversity and body mass index in childhood and adolescence: Linking registry data on adversities with school health records of 53,401 children from Copenhagen. International Journal of Obesity, 47(11), 1057–1064. 10.1038/s41366-023-01355-9 PMC10599995 37626127

[bibr32-15248380251383934] Felitti V. J. Anda R. F. Nordenberg D. Williamson D. F. Spitz A. M. Edwards V. Koss M. P. Marks J. S. (1998). Relationship of childhood abuse and household dysfunction to many of the leading causes of death in adults: The Adverse Childhood Experiences (ACE) study. American Journal of Preventive Medicine, 14(4), 245–258. 10.1016/S0749-3797(98)00017-8 9635069

[bibr33-15248380251383934] Fitzpatrick T. McNally J. D. Stukel T. A. Lu H. Fisman D. Kwong J. C. Guttmann A. (2021). Family and child risk factors for early-life RSV illness. Pediatrics (Evanston), 147(4), e2020029090. 10.1542/peds.2020-029090 33737374

[bibr34-15248380251383934] Flack F. Kemp-Casey A. Wray N. (2019). Using linked administrative data in clinical trials: A guide for clinical trialists and researchers. https://clinicaltrialsalliance.org.au/wp-content/uploads/2019/09/Linked-data-guidance_final.pdf

[bibr35-15248380251383934] Fleming M. McLay J. S. Clark D. King A. Mackay D. F. Minnis H. Pell J. P. (2021). Educational and health outcomes of schoolchildren in local authority care in Scotland: A retrospective record linkage study. PLoS Medicine, 18(11), e1003832. 10.1371/journal.pmed.1003832 PMC8589203 34767555

[bibr36-15248380251383934] Font S. A. Cancian M. Berger L. M. (2019). Prevalence and risk factors for early motherhood among low-Income, maltreated, and foster youth. Demography, 56(1), 261–284. 10.1007/s13524-018-0744-x PMC6396831 30519845

[bibr37-15248380251383934] Ford K. Butler N. Hughes K. Quigg Z. Bellis M. A. (2016). Adverse Childhood Experiences (ACEs) in Hertfordshire, Luton and Northamptonshire. Centre for Public Health, Liverpool John Moores University.

[bibr38-15248380251383934] Ford K. Hughes K. Hardcastle K. Di Lemma L. C. G. Davies A. R. Edwards S. Bellis M. A. (2019). The evidence base for routine enquiry into adverse childhood experiences: A scoping review. Child Abuse & Neglect, 91, 131–146. 10.1016/j.chiabu.2019.03.007 30884399

[bibr39-15248380251383934] Francis E. R. Tsaligopoulou A. Stock S. E. Pingault J. B. Baldwin J. R. (2023). Subjective and objective experiences of childhood adversity: A meta-analysis of their agreement and relationships with psychopathology. Journal of Child Psychology and Psychiatry, 64(8), 1185–1199. 10.1111/jcpp.13803 PMC10617978 37186463

[bibr40-15248380251383934] Gnanamanickam E. S. Nguyen H. Armfield J. M. Doidge J. C. Brown D. S. Preen D. B. Segal L. (2020). Hospitalizations among children involved in the child protection system: A long-term birth cohort study from infancy to adulthood using administrative data. Child Abuse & Neglect, 107, 104518–104518. 10.1016/j.chiabu.2020.104518 32652507

[bibr41-15248380251383934] Han E. Kharrazi H. Shi L. (2023). Identifying predictors of nursing home admission by using electronic health records and administrative data: Scoping review. JMIR Aging, 6, e42437–e42437. 10.2196/42437 PMC10686617 37990815

[bibr42-15248380251383934] Hardt J. Rutter M. (2004). Validity of adult retrospective reports of adverse childhood experiences: Review of the evidence. Journal of Child Psychology and Psychiatry, 45(2), 260–273. 10.1111/j.1469-7610.2004.00218.x 14982240

[bibr43-15248380251383934] Harris L. G. Higgins D. J. Willis M. L. Lawrence D. Meinck F. Thomas H. J. Malacova E. Scott J. G. Pacella R. Haslam D. M. (2024). Dimensions of child maltreatment in Australians with a history of out-of-home care. Child Maltreatment, 30(3), 525–539. 10.1177/10775595241297944 PMC12144324 39499703

[bibr44-15248380251383934] Harron K. Dibben C. Boyd J. Hjern A. Azimaee M. Barreto M. L. Goldstein H. (2017). Challenges in administrative data linkage for research. Big Data & Society, 4(2), 1–12. 10.1177/2053951717745678 PMC6187070 30381794

[bibr45-15248380251383934] Hash J. B. Oxford M. L. Ward T. M. Fleming C. B. Spieker S. J. (2020). Sleep patterns, problems and ecology among toddlers in families with a child protective services maltreatment referral. Journal of Pediatric Nursing, 51, 85–91. 10.1016/j.pedn.2019.12.015 PMC7276301 31945664

[bibr46-15248380251383934] Hasson R. G. Crea T. M. Easton S. D. Underwood D. (2022). Child maltreatment and substance abuse: Unaccompanied children and adverse outcomes in long-term foster care in the United States. Child & Family Social Work, 27(3), 435–443. 10.1111/cfs.12895

[bibr47-15248380251383934] He V. Y. Leckning B. Malvaso C. Williams T. Liddle L. Guthridge S. (2021). Opportunities for prevention: a data-linkage study to inform a public health response to youth offending in the Northern Territory, Australia. BMC Public Health, 21(1), 1–1600. 10.1186/s12889-021-11645-4 PMC8404303 34461862

[bibr48-15248380251383934] Henry B. F. (2020). Typologies of adversity in childhood & adulthood as determinants of mental health & substance use disorders of adults incarcerated in US prisons. Child Abuse & Neglect, 99, 104251–104251. 10.1016/j.chiabu.2019.104251 PMC6937387 31726246

[bibr49-15248380251383934] Hinnen C. von Haeseler E. Tijssens F. Mols F. (2024). Adverse childhood events and mental health problems in cancer survivors: A systematic review. Supportive Care in Cancer, 32(1), 80–80. 10.1007/s00520-023-08280-7 PMC10766658 38175303

[bibr50-15248380251383934] Jaen J. Lovett S. M. Lajous M. Keyes K. M. Stern D. (2023). Adverse childhood experiences and adult outcomes using a causal framework perspective: Challenges and opportunities. Child Abuse & Neglect, 143, 106328–106328. 10.1016/j.chiabu.2023.106328 37379730

[bibr51-15248380251383934] Jay M. A. Grath-Lone L. M. De Stavola B. Gilbert R. (2022). Evaluation of pushing out of children from all English state schools: Administrative data cohort study of children receiving social care and their peers. Child Abuse & Neglect, 127, 105582. 10.1016/j.chiabu.2022.105582 PMC9077441 35255340

[bibr52-15248380251383934] Jonson-Reid M. Presnall N. Drake B. Fox L. Bierut L. Reich W. Kane P. Todd R. D. Constantino J. (2010). Effects of child maltreatment and inherited liability on antisocial development: An official records study. Journal of the American Academy of Child and Adolescent Psychiatry, 49(4), 321–332. 10.1016/j.jaac.2009.11.015 PMC2878182 20410725

[bibr53-15248380251383934] Jutte D. P. Roos L. L. Brownell M. D. (2011). Administrative record linkage as a tool for public health research. Annual Review of Public Health, 32(1), 91–108. 10.1146/annurev-publhealth-031210-100700 21219160

[bibr54-15248380251383934] Karatekin C. Hill M. (2018). Expanding the original definition of Adverse Childhood Experiences (ACEs). Journal of Child & Adolescent Trauma, 12(3), 289–306. 10.1007/s40653-018-0237-5 PMC7163861 32318200

[bibr55-15248380251383934] Karatekin C. Mason S. M. Riegelman A. Bakker C. Hunt S. Gresham B. Corcoran F. Barnes A. (2022). Adverse childhood experiences: A scoping review of measures and methods. Children and Youth Services Review, 136, 106425. 10.1016/j.childyouth.2022.106425

[bibr56-15248380251383934] Karriker-Jaffe K. J. Lönn S. L. Cook W. K. Kendler K. S. Sundquist K. (2018). Chains of risk for alcohol use disorder: Mediators of exposure to neighborhood deprivation in early and middle childhood. Health & Place, 50, 16–26. 10.1016/j.healthplace.2017.12.008 PMC5834358 29334617

[bibr57-15248380251383934] Kedzior S. G. E. Barrett S. Muir C. Lynch R. Kaner E. Forman J. R. Wolfe I. McGovern R. (2024). “They had clothes on their back and they had food in their stomach, but they didn’t have me”: The contribution of parental mental health problems, substance use, and domestic violence and abuse on young people and parents. Child Abuse & Neglect, 149, 106609. 10.1016/j.chiabu.2023.106609 38181566

[bibr58-15248380251383934] Lacey R. E. Pinto Pereira S. M. Li L. Danese A. (2020). Adverse childhood experiences and adult inflammation: Single adversity, cumulative risk and latent class approaches. Brain, Behavior, and Immunity, 87, 820–830. 10.1016/j.bbi.2020.03.017 PMC7327510 32201253

[bibr59-15248380251383934] Leckning B. Condon J. R. Das S. K. He V. Hirvonen T. Guthridge S. (2023). Mental health-related hospitalisations associated with patterns of child protection and youth justice involvement during adolescence: A retrospective cohort study using linked administrative data from the Northern Territory of Australia. Children and Youth Services Review, 145, 106771. 10.1016/j.childyouth.2022.106771

[bibr60-15248380251383934] Lee M. Y. Jonson-Reid M. (2009). Needs and outcomes for low income youth in special education: Variations by emotional disturbance diagnosis and child welfare contact. Children and Youth Services Review, 31(7), 722–731. 10.1016/j.childyouth.2009.01.005 PMC2732122 19714226

[bibr61-15248380251383934] Loxton D. Townsend N. Dolja-Gore X. Forder P. Coles J. (2019). Adverse childhood experiences and healthcare costs in adult life. Journal of Child Sexual Abuse, 28(5), 511–525. 10.1080/10538712.2018.1523814 30285583

[bibr62-15248380251383934] Lut I. Harron K. Hardelid P. O’Brien M. Woodman J. (2022). ‘What about the dads?’ Linking fathers and children in administrative data: A systematic scoping review. Big Data & Society, 9(1), 205395172110692. 10.1177/20539517211069299

[bibr63-15248380251383934] Malvaso C. Montgomerie A. Pilkington R. M. Baker E. Lynch J. W. (2022). Examining the intersection of child protection and public housing: development, health and justice outcomes using linked administrative data. BMJ Open, 12(6), e057284. 10.1136/bmjopen-2021-057284 PMC9189815 35688602

[bibr64-15248380251383934] Matsuura N. Hashimoto T. Toichi M. (2013). Associations among adverse childhood experiences, aggression, depression, and self-esteem in serious female juvenile offenders in Japan. The Journal of Forensic Psychiatry & Psychology, 24(1), 111–127. 10.1080/14789949.2012.746384

[bibr65-15248380251383934] Mc Grath-Lone L. Jay M. A Blackburn R Gordon E Zylbersztejn A. Wiljaars L Gilbert R . (2022). What makes administrative data “research-ready”? A systematic review and thematic analysis of published literature. International Journal of Population Data Science, 7(1), 1718. 10.23889/IJPDS.V7I1.1718 PMC9052961 35520099

[bibr66-15248380251383934] McCutcheon V. V. Bucholz K. K. Houston-Ludlam A. Heath A. C. (2019). Elevated maternal and child mortality among women with multiple DUIs compared to sociodemographically matched Controls. Addiction (Abingdon, England), 114(11), 1981–1991. 10.1111/add.14762 PMC6800795 31351443

[bibr67-15248380251383934] Melis G. Bedston S. Akbari A. Bennett D. Lee A. Lowthian E. Schlüter D. Taylor—Robinson D. (2023). Impact of socio-economic conditions and perinatal factors on risk of becoming a child looked after: A whole population cohort study using routinely collected data in Wales. Public Health (London), 224, 215–223. 10.1016/j.puhe.2023.09.001 37856904

[bibr68-15248380251383934] Meurk C. Wittenhagen L. Steele M. L. Ferris L. Edwards B. Bosley E. Heffernan E. (2021). Examining the use of police and ambulance data in suicide Research A systematic scoping review of data linkage studies. Crisis: The Journal of Crisis Intervention and Suicide Prevention, 42(5), 386–395. 10.1027/0227-5910/a000739 33241741

[bibr69-15248380251383934] Miyamoto S. Romano P. S. Putnam-Hornstein E. Thurston H. Dharmar M. Joseph J. G. (2017). Risk factors for fatal and non-fatal child maltreatment in families previously investigated by CPS: A case-control study. Child Abuse & Neglect, 63, 222–232. 10.1016/j.chiabu.2016.11.003 27881240

[bibr70-15248380251383934] National Bureau of Economic Research. (2024). Survey of Epidemiology and End Results (SEER) U.S. State and county population data by age, race, sex, Hispanic 1969-on. National Bureau of Economic Research. https://www.nber.org/research/data/survey-epidemiology-and-end-results-seer-us-state-and-county-population-data-age-race-sex-hispanic

[bibr71-15248380251383934] National Center for Injury Prevention and Control, Centers for Disease Control and Prevention. (2021). Adverse Childhood Experiences prevention strategy. https://dam.assets.ohio.gov/image/upload/preventioncoe.ohio.gov/HealthEquity/CDC-ACEs-Prevention-Strategy.pdf

[bibr72-15248380251383934] Neagoe I. M. Papasteri C. C. (2023). Adverse Childhood Experiences in residential care: Predicting accumulation and exploring clustering of adversity in Romanian care facilities. Child & Youth Care Forum, 52(4), 779–800. 10.1007/s10566-022-09712-x

[bibr73-15248380251383934] Negriff S. Schneiderman J. U. Trickett P. K. (2017). Concordance between self-reported childhood maltreatment versus case record reviews for child welfare–affiliated adolescents: Prevalence rates and associations with outcomes. Child Maltreatment, 22(1), 34–44. 10.1177/1077559516674596 PMC5353974 27777329

[bibr74-15248380251383934] Nikulina V. Gelin M. Zwilling A. (2021). Is there a cumulative association between Adverse Childhood Experiences and intimate partner violence in emerging adulthood? Journal of Interpersonal Violence, 36(3–4), NP1205–NP1232. 10.1177/0886260517741626 29295018

[bibr75-15248380251383934] Nilsson E. Hultman C. M. Cnattingius S. Olausson P. O. Björk C. Lichtenstein P. (2008). Schizophrenia and offspring’s risk for adverse pregnancy outcomes and infant death. British Journal of Psychiatry, 193(4), 311–315. 10.1192/bjp.bp.107.045146 18827293

[bibr76-15248380251383934] O’Donnell M. Maclean M. J. Sims S. Morgan V. A. Leonard H. Stanley F. J. (2015). Maternal mental health and risk of child protection involvement: Mental health diagnoses associated with increased risk. Journal of Epidemiology and Community Health, 69(12), 1175–1183. 10.1136/jech-2014-205240 26372788

[bibr77-15248380251383934] O’Hare K. Watkeys O. Whitten T. Dean K. Laurens K. R. Harris F. Carr V. J. Green M. J. (2022). Parental and community risk factors for childhood self-harm thoughts and behaviours. Journal of Affective Disorders, 310, 279–283. 10.1016/j.jad.2022.05.050 35569604

[bibr78-15248380251383934] O’Leary C. Lawrence D. Hafekost K. Zubrick S. R. Bower C. (2020). Maternal alcohol-use disorder and child outcomes. Pediatrics (Evanston), 145(3), e20191574. 10.1542/peds.2019-1574 32094291

[bibr79-15248380251383934] Orr C. Fisher C. Bell M. O’Donnell M. Martin K. Glauert R. Preen D. (2023). Exposure to family and domestic violence is associated with lower attendance and higher suspension in school children. Child Abuse and Neglect, 142, 105594. 10.1016/j.chiabu.2022.105594 35459527

[bibr80-15248380251383934] Orr C. Fisher C. Sims S. Preen D. Glauert R. O’Donnell M. (2019). Hospitalisations for maternal assault are associated with increased risk of child protection involvement. Child Abuse & Neglect, 95, 104014–104014. 10.1016/j.chiabu.2019.05.007 31325682

[bibr81-15248380251383934] Orr C. Fisher C. M. Preen D. B. Glauert R. A. O’Donnell M. (2020). Exposure to family and domestic violence is associated with increased childhood hospitalisations. PLoS One, 15(8), e0237251. 10.1371/journal.pone.0237251 PMC7413507 32764798

[bibr82-15248380251383934] Panteli D. Polin K. Webb E. Allin S. Barnes A. Degelsegger-Márquez A. Ghafur S. Jamieson M. Kim Y. Litvinova Y. Nimptsch U. Parkkinen M. Rasmussen T. A. Reichebner C. Röttger J. Rumball-Smith J. Scarpetti G. Seidler A. L. Seppänen J. Smith M. Snell M. Stanimirovic D. Verheij R. Zaletel M. Busse R. (2023). Health and care data: Approaches to data linkage for evidence-informed policy. Health Systems in Transition, 25(2), 1–248. https://iris.who.int/bitstream/handle/10665/371097/9789289059466-eng.pdf?sequence=1 37489953

[bibr83-15248380251383934] Pattaro S. Bailey N. Dibben C. (2020). Using linked longitudinal administrative data to identify social disadvantage. Social Indicators Research, 147(3), 865–895. 10.1007/s11205-019-02173-1

[bibr84-15248380251383934] Rahapsari S. Levita L. (2024). The impact of Adverse Childhood Experiences on cognitive control across the lifespan: A systematic review and meta-analysis of prospective studies. Trauma, Violence, & Abuse, 26(4), 712–733. 10.1177/15248380241286812 PMC12397554 39396188

[bibr85-15248380251383934] Ranning A. Uddin M. J. Sørensen H. J. Laursen T. M. Thorup A. A. E. Madsen T. Nordentoft M. Erlangsen A. (2022). Intergenerational transmission of suicide attempt in a cohort of 4.4 million children. Psychological Medicine, 52(14), 3202–3209. 10.1017/S0033291720005310 33827720

[bibr86-15248380251383934] Regmi S. Kedia S. K. Ahuja N. A. Lee G. Entwistle C. Dillon P. J. (2023). Association between adverse childhood experiences and opioid use-related behaviors: A systematic review. Trauma, Violence, & Abuse, 25(3), 2046–2064. 10.1177/15248380231205821 37920999

[bibr87-15248380251383934] Reuben A. Moffitt T. E. Caspi A. Belsky D. W. Harrington H. Schroeder F. Hogan S. Ramrakha S. Poulton R. Danese A. (2016). Lest we forget: Comparing retrospective and prospective assessments of adverse childhood experiences in the prediction of adult health. Journal of Child Psychology and Psychiatry, 57(10), 1103–1112. 10.1111/jcpp.12621 PMC5234278 27647050

[bibr88-15248380251383934] Ringbäck Weitoft G Hjern A Batljan I. Vinnerljung B . (2008). Health and social outcomes among children in low-income families and families receiving social assistance: A Swedish national cohort study. Social Science & Medicine, 66(1), 14–30. 10.1016/j.socscimed.2007.07.031 17889976

[bibr89-15248380251383934] Sasaki N. Watanabe K. Kanamori Y. Tabuchi T. Fujiwara T. Nishi D. (2024). Effects of expanded adverse childhood experiences including school bullying, childhood poverty, and natural disasters on mental health in adulthood. Scientific Reports, 14(1), 12015. 10.1038/s41598-024-62634-7 PMC11128446 38797740

[bibr90-15248380251383934] Sims S. O’Donnell M. (2015). Identifying families most likely to have missing paternal details in birth registrations using linked data. Journal of Family Studies, 21(2), 163–177. 10.1080/13229400.2015.1020983

[bibr91-15248380251383934] Smith J. N. Guttmann A. Kopp A. Vandermorris A. Shouldice M. Harron K. L. (2024). Association of maternal risk factors with infant maltreatment: An administrative data cohort study. Archives of Disease in Childhood, 109(1), 23–29. 10.1136/archdischild-2022-325239 37758290

[bibr92-15248380251383934] SmithBattle L. Loman D. G. Yoo J. H. Cibulka N. Rariden C. (2022). Evidence for revising the Adverse Childhood Experiences screening tool: A scoping review. Journal of Child & Adolescent Trauma, 15(1), 89–103. 10.1007/s40653-021-00358-w PMC8837767 35222777

[bibr93-15248380251383934] Statistics Canada. (2023). Administrative data. https://www.statcan.gc.ca/en/our-data/where/administrative-data

[bibr94-15248380251383934] Stewart A. Livingston M. Dennison S. (2008). Transitions and turning points: Examining the links between child maltreatment and juvenile offending. Child Abuse & Neglect, 32(1), 51–66. 10.1016/j.chiabu.2007.04.011 18082884

[bibr95-15248380251383934] Toivonen K. Salokekkilä P. Puustelli A. Häggman-Laitila A. (2020). Somatic and mental symptoms, medical treatments and service use in aftercare—Document analysis of Finnish care leavers. Children and Youth Services Review, 114, 105079. 10.1016/j.childyouth.2020.105079

[bibr96-15248380251383934] Tzouvara V. Kupdere P. Wilson K. Matthews L. Simpson A. Foye U. (2023). Adverse childhood experiences, mental health, and social functioning: A scoping review of the literature. Child Abuse & Neglect, 139, 106092–106092. 10.1016/j.chiabu.2023.106092 36907117

[bibr97-15248380251383934] Valuri G. M. Morgan F. Jablensky A. Morgan V. A. (2017). Impact of social disadvantage and parental offending on rates of criminal offending among offspring of women with severe mental illness. Australian and New Zealand Journal of Psychiatry, 51(10), 1032–1040. 10.1177/0004867416688099 28093927

[bibr98-15248380251383934] Walsh M. C. Joyce S. Maloney T. Vaithianathan R. (2020). Exploring the protective factors of children and families identified at highest risk of adverse childhood experiences by a predictive risk model: An analysis of the growing up in New Zealand cohort. Children & Youth Services Review, 108. 10.1016/j.childyouth.2019.104556

[bibr99-15248380251383934] Whitten T. Green M. J. Laurens K. R. Tzoumakis S. Harrs F. Carr V. Dean K. (2019). Parental offending and children’s emergency department presentations in New South Wales, Australia. Journal of Epidemiology and Community Health, 73(9), 832–838. 10.1136/jech-2019-212392 31152075

[bibr100-15248380251383934] Woollard M. (2014). Administrative data: Problems and benefits. A perspective from the United Kingdom. In Duşa A. Nelle D. Stock G. Wagner G. (Eds.), Facing the Future: European Research Infrastructures for the Humanities and Social Science (1st ed., pp. 49–60). SCIVERO Verlag.

[bibr101-15248380251383934] World Bank Group. (2025). Statistical Performance Indicators (SPI). World Bank Group. https://www.worldbank.org/en/programs/statistical-performance-indicators

[bibr102-15248380251383934] Yamana H. Konishi T. Yasunaga H. (2023). Validation studies of Japanese administrative health care data: A scoping review. Pharmacoepidemiology and Drug Safety, 32(7), 705–717. 10.1002/pds.5636 37146098

[bibr103-15248380251383934] Yampolskaya S. Armstrong M. I. McNeish R. (2011). Children placed in out-of-home care: Risk factors for involvement with the juvenile justice system. Violence and Victims, 26(2), 231–245. 10.1891/0886-6708.26.2.231 21780537

[bibr104-15248380251383934] Young A. Flack F. (2018). Recent trends in the use of linked data in Australia. Australian Health Review, 42(5), 584–590. 10.1071/AH18014 30145995

[bibr105-15248380251383934] Zhang L. Mersky J. P. Gruber A. M. H. Kim J.-Y. (2023). Intergenerational transmission of parental Adverse Childhood Experiences and children’s outcomes: A scoping review. Trauma, Violence, & Abuse, 24(5), 3251–3264. 10.1177/15248380221126186 36205317

