# Supplementary material for: The Use of Linked Administrative Data to Examine the Impact of Adverse Childhood Experiences: A Scoping Review of the Literature
Source: Trauma Violence Abuse. 2025 Nov 19;27(5):2544–61. doi: 10.1177/15248380251383934 (PMC13736288; doi:10.1177/15248380251383934)
Supplement: Supplementary file 1 [file sj-docx-1-tva-10.1177_15248380251383934.docx]

Supplementary Table 1 – Complete list of search terms

| ***Database*** | ***Adverse Childhood Experience Subject Heading*** | ***Adverse Childhood Experience***  ***Search Terms and Syntax*** | ***Linked Administrative Data*** |
| --- | --- | --- | --- |
| Ovid – Medline, PsycINFO, Embase, | Medline – Adverse Childhood Experiences  PsycINFO – Childhood Adversity  Embase – Childhood Adversity | ***New:***  Adverse childhood experience* OR problematic childhood OR  (early life* ADJ3 (trauma* OR advers* OR sorrow OR stress OR misfortune OR maltreat* OR neglect OR abuse OR rape)) OR  (child* ADJ3 (trauma* OR advers* OR sorrow OR stress OR misfortune OR maltreat* OR neglect OR abuse OR rape)) OR  (adolescen* ADJ3 (trauma* or advers* OR sorrow OR stress OR misfortune OR maltreat* OR neglect OR abuse OR rape)) OR  (Parent* OR mother OR father OR maternal OR paternal) ADJ3 (death OR mental ill* OR depression OR anxiety OR psych* OR substance OR drug OR alcohol OR addict OR incarcerat* OR arrest OR crim* OR jail* OR prison) OR  domestic violence OR IPV OR intimate partner violence OR DV OR FDV OR domestic abuse OR marital violence OR gender* violence OR family dysfunction OR household dysfunction OR poverty OR disadvantage OR divorce | administrat* data OR linked data OR routinely collected data OR population level data OR electronic records OR administrative claims data OR data linked to other sources OR health care data OR registry data OR population registry OR record* link* OR data link* OR population data |
| Proquest – ERIC | ERIC – | Adverse childhood experience* OR problematic childhood OR  (early life* NEAR/3 (trauma* OR advers* OR sorrow OR stress OR misfortune OR maltreat* OR neglect OR abuse OR rape)) OR  (child* NEAR/3 (trauma* OR advers* OR sorrow OR stress OR misfortune OR maltreat* OR neglect OR abuse OR rape)) OR  (adolescen* NEAR/3 (trauma* or advers* OR sorrow OR stress OR misfortune OR maltreat* OR neglect OR abuse OR rape)) OR  (parent* OR mother OR father OR maternal OR paternal) NEAR/3 (death OR “mental ill*” OR depression OR anxiety OR psych* OR substance OR drug OR alcohol OR addict OR incarcerat* OR arrest OR crim* OR jail* OR prison) OR  domestic violence OR IPV OR intimate partner violence OR DV OR FDV OR domestic abuse OR marital violence OR gender* violence OR family dysfunction OR household dysfunction OR poverty OR disadvantage OR divorce | “administrat* data” OR “linked data” OR “routinely collected data” OR “population level data” OR “electronic records” OR “administrative claims data” OR “data linked to other sources” OR “health care data” OR “registry data” OR “population registry” OR “record* link*” OR “data link*” OR “population data” |
| EBSCO – Education Source  CINAHL Plus | Education Source –  CINAHL Plus – | Adverse childhood experience* OR problematic childhood OR  (early life* N3 (trauma* OR advers* OR sorrow OR stress OR misfortune OR maltreat* OR neglect OR abuse OR rape)) OR  (child* N3 (trauma* OR advers* OR sorrow OR stress OR misfortune OR maltreat* OR neglect OR abuse OR rape)) OR  (adolescen* N3 (trauma* or advers* OR sorrow OR stress OR misfortune OR maltreat* OR neglect OR abuse OR rape)) OR  (Parent* OR mother OR father OR maternal OR paternal) N3 (death OR mental ill* OR depression OR anxiety OR psych* OR substance OR drug OR alcohol OR addict OR incarcerat* OR arrest OR crim* OR jail* OR prison) OR  domestic violence OR IPV OR intimate partner violence OR DV OR FDV OR domestic abuse OR marital violence OR gender* violence OR family dysfunction OR household dysfunction OR poverty OR disadvantage OR divorce | “administrat* data” OR “linked data” OR “routinely collected data” OR “population level data” OR “electronic records” OR “administrative claims data” OR “data linked to other sources” OR “health care data” OR “registry data” OR “population registry” OR “record* link*” OR “data link*” OR “population data” |
