# Supplementary material for: The Use of Linked Administrative Data to Examine the Impact of Adverse Childhood Experiences: A Scoping Review of the Literature
Source: Trauma Violence Abuse. 2025 Nov 19;27(5):2544–61. doi: 10.1177/15248380251383934 (PMC13736288; doi:10.1177/15248380251383934)
Supplement: Supplementary file 2 [file sj-docx-2-tva-10.1177_15248380251383934.docx]

| **Year** | **Study/Paper Title** | **Reference** | **Sample Size** | **Location** | **Age of Exposure** | **Exposure** | **Outcome** |
| --- | --- | --- | --- | --- | --- | --- | --- |
| 1998 | Child abuse and severity of disturbance among adult psychiatric inpatients | (Read, 1998) | 100 | New Zealand | Child | Physical, Sexual | Health |
| 1999 | The status of older adolescents at exit from out-of-home care | (McMillen & Tucker, 1999) | 252 | USA  (Missouri) | Child | Physical, Psychological, Sexual | Welfare, Education |
| 2000 | Children in foster care: factors influencing outpatient mental health service use | (Leslie et al., 2000) | 480 | USA  (San Diego) | 1-3 yrs  4-5 yrs  6-11 yrs  12-17 yrs | Physical, Psychological, Sexual | Health |
| 2000 | From maltreatment report to juvenile incarceration: the role of child welfare services | (Jonson-Reid & Barth, 2000) | 159,549 | USA  (California) | 7-11 yrs  12-14 yrs  14-17 yrs | Physical, Psychological, Sexual | Justice |
| 2001 | Adolescent sexual offenders: Incidence of childhood maltreatment, serious emotional disturbance, and prior offenses | (Jonson-Reid & Way, 2001) | 6,082 | USA  (California) | 11-18 yrs | Physical, Psychological, Sexual | Justice |
| 2001 | Multivariate impact of health and caretaking risk factors on the school adjustment of first graders | (Weiss & Fantuzzo, 2001) | 9,088 | USA | Birth to First Grade | Physical, Psychological, Sexual | Education |
| 2001 | Self-Sufficiency of Former Foster Youth in Wisconsin: Analysis of Unemployment Insurance Wage Data and Public Assistance Data. Special Report | (Dworsky & Courtney, 2001) | 6,274 | USA  (Wisconsin) | Age at most recent episode  7-9 yrs  10-11 yrs  12-13 yrs  14-15 yrs  16-17 yrs | Physical, Psychological, Sexual | Welfare |
| 2002 | The risk of subsequent maltreatment allegations in families with substance-exposed infants | (Smith & Testa, 2002) | 277 | USA  (Illinois) | Prenatal | Substance Abuse | Welfare |
| 2003 | Cross-type recidivism among child maltreatment victims and perpetrators | (Jonson-Reid et al., 2003) | 61,693 | USA  (Missouri) | Child | Physical, Psychological, Sexual | Welfare |
| 2004 | Impact of child sexual abuse on mental health: prospective study in males and females | (Spataro et al., 2004) | 1,612 | Australia  (Victoria) | 16 years of age and younger | Sexual | Health |
| 2005 | Maternal and Neonatal Outcomes of Assaults During Pregnancy | (El Kady et al., 2005) | 2,070 | USA  (California) | Prenatal | Domestic Violence | Health |
| 2006 | Re-reporting of child maltreatment: does participation in other public sector services moderate the likelihood of a second maltreatment report? | (Drake et al., 2006) | 4,957 | USA (Midwestern metropolitan area) | Birth – 11 yrs | Physical, Psychological, Sexual, Mental Illness, Substance Abuse | Welfare |
| 2006 | Use of record linkage to examine alcohol use in pregnancy | (Burns et al., 2006) | 416,834 | Australia  (New South Wales) | Prenatal | Substance Abuse | Health |
| 2006 | Where are we now?: A post-ASFA examination of adoption disruption | (Smith et al., 2006) | 15,947 | USA | 0-2 yrs  2-3 yrs  4-6 yrs  7-9 yrs  10-12 yrs  13+ yrs | Physical, Psychological, Sexual | Welfare |
| 2007 | An exploratory study of drug-exposed infants: case substantiation and subsequent child maltreatment | (Sun et al., 2007) | 457 | USA  (Nevada) | Infant | Substance Abuse | Welfare |
| 2007 | Educational Outcomes of Children on Guardianship or Custody Orders: A Pilot Study. | (Australian Institute of Health and Welfare, 2007) | 895 | Australia (Victoria, Queensland, South Australia, Tasmania, Australian Capital Territory) | Children in Years 3, 5 and 7 | Physical, Psychological, Sexual | Education |
| 2007 | Foster children with an incarcerated parent: Predictors of reunification | (Hayward & DePanfilis, 2007) | 40,751 | USA | 0-2 yrs  3-5 yrs  6-12 yrs  13-17 yrs  17-20 yrs | Physical, Psychological, Sexual, Substance Abuse, Criminal Behaviour | Welfare |
| 2008 | Familial clustering of suicidal behaviour and psychopathology in young suicide attempters. A register-based nested case control study | (Mittendorfer-Rutz et al., 2008) | 14,440 | Sweden | 10 yrs – end follow up | Mental Illness, Substance Abuse | Health |
| 2008 | Health and social outcomes among children in low-income families and families receiving social assistance--a Swedish national cohort study | (Ringbäck Weitoft et al., 2008) | 1,200,000 | Sweden | 0-15 yrs (cohort)  3-18 yrs (start follow up)  14-27 yrs (end study) | Mental Illness, Substance Abuse | Health, Education, Welfare |
| 2008 | Schizophrenia and offspring's risk for adverse pregnancy outcomes and infant death | (Nilsson et al., 2008) | 1,890,550 | Sweden | Neonatal (0-27 days),  Infant (0-364 days) | Mental Illness | Health |
| 2008 | Transitions and turning points: examining the links between child maltreatment and juvenile offending | (Stewart et al., 2008) | 5,849 | Australia (Queensland) | Birth-5 yrs  6-11 yrs  12-17 yrs  Both childhood and adolescence | Physical, Psychological, Sexual | Justice |
| 2009 | Characteristics of clients to a suicide prevention centre--an epidemiological analysis of the users over a 10-year period | (Qin et al., 2009) | 3,505 | Denmark | 9-20 yrs old (and adult ages) | Mental Illness | Health |
| 2009 | Increased number of offspring in first degree relatives of psychotic individuals: a partial explanation for the persistence of psychotic illnesses | (Weiser et al., 2009) | 4,904 | Israel | Child | Mental Illness | Health |
| 2009 | Needs and outcomes for low income youth in special education: Variations by emotional disturbance diagnosis and child welfare contact | (Lee & Jonson-Reid, 2009) | 471 | USA | Child | Physical, Psychological, Sexual | Health, Education, Justice |
| 2010 | Characteristics of non-Aboriginal and Aboriginal children and families with substantiated child maltreatment: a population based study | (M O'Donnell, N Nassar, H Leonard, P Jacoby, et al., 2010) | 6,486 | Australia  (Western Australia) | Child | Mental Illness, Substance Abuse, Domestic Violence | Welfare |
| 2010 | Child maltreatment and pediatric health outcomes: a longitudinal study of low-income children | (Lanier et al., 2010) | 6,282 | USA (Midwestern metropolitan region) | 0-11 yrs | Physical, Psychological, Sexual | Health |
| 2010 | Effects of Child Maltreatment and Inherited Liability on Antisocial Development: An Official Records Study | (M Jonson-Reid et al., 2010) | 4,432 | USA  (Missouri) | Child | Physical, Psychological, Sexual | Health, Justice |
| 2010 | Infant maltreatment-related mortality in Alaska: Correcting the count and using birth certificates to predict mortality | (Parrish & Gessner, 2010) | 955 | USA  (Alaska) | Infant | Substance Abuse | Health |
| 2010 | Infants in Australian out-of-home care | (Zhou & Chilvers, 2010) | 5,738 | Australia  (New South Wales) | Infant | Physical, Psychological, Sexual, Substance Abuse, Domestic Violence, Mental Illness | Welfare |
| 2010 | Monitoring child abuse and neglect at a population level: patterns of hospital admissions for maltreatment and assault | (M O'Donnell, N Nassar, H Leonard, R Mathews, et al., 2010) | 6,191 | Australia  (Western Australia) | 0-17 yrs | Physical, Psychological, Sexual | Health |
| 2010 | Psychopathology in a large cohort of sexually abused children followed up to 43 years | (M. C. Cutajar et al., 2010) | 2,759 | Australia  (Victoria) | ≤16 yrs | Sexual | Health |
| 2010 | Rates and types of hospitalisations for children who have subsequent contact with the child protection system: a population based case-control study | (M. O'Donnell et al., 2010) | 20,134 | Australia  (Western Australia) | 0-15 yrs | Physical, Psychological, Sexual | Health |
| 2010 | Schizophrenia and other psychotic disorders in a cohort of sexually abused children | (M. Cutajar et al., 2010) | 2,759 | Australia  (Victoria) | <16 yrs | Sexual | Health |
| 2010 | The use of cross-jurisdictional population data to investigate health indicators of child maltreatment | (Melissa O'Donnell et al., 2010) | 397,346 | Australia (Western Australia) | 0-5 yrs  6-11 yrs  12-15 yrs | Physical, Psychological, Sexual | Welfare, Health |
| 2010 | Timing and influence of early experiences of child maltreatment and homelessness on children's educational well-being | (Perlman & Fantuzzo, 2010) | 12,045 | USA  (Northeastern County) | Up to second grade (av age 8.5 yrs) | Physical, Psychological, Sexual | Education |
| 2010 | Understanding chronically reported families | (Melissa Jonson-Reid et al., 2010) | 6,365 | USA  (Midwest metropolitan area) | Child to 18 | Physical, Psychological, Sexual, Mental Illness | Welfare |
| 2011 | Children placed in out-of-home care: risk factors for involvement with the juvenile justice system | (Yampolskaya, Armstrong, & McNeish, 2011) | 13,212 | USA  (Florida) | 7 to 17 | Physical, Psychological, Sexual | Justice |
| 2011 | Contextual and individual-level predictors of abused children’s reentry into out-of-home care: A multilevel mixture survival analysis | (Yampolskaya, Armstrong, & King-Miller, 2011) | 17,695 | USA  (Florida) | 1 month to 20 years | Physical, Psychological, Sexual, Substance Abuse | Welfare |
| 2011 | Epidemiology of attempted suicide in Hatay, Turkey | (Turhan et al., 2011) | 1,613 | Turkey | <15  15-24  Adult ages | Mental Illness | Health |
| 2011 | Filicide in Offspring of Parents With Severe Psychiatric Disorders: A Population-Based Cohort Study of Child Homicide | (Laursen et al., 2011) | 187 | Denmark | Birth to 18 years | Mental Illness | Health |
| 2011 | Geographic and Maternal Characteristics Associated with Alcohol Use in Pregnancy | (Burns et al., 2011) | 417,464 | Australia  (New South Wales) | Prenatal and birth | Substance Abuse | Education |
| 2011 | Maternal and foetal outcomes among pregnant women hospitalised due to interpersonal violence: a population-based study in Western Australia, 2002-2008 | (Meuleners et al., 2011) | 468 | Australia  (Western Australia) | Foetus and infant | Domestic Violence | Health |
| 2011 | Report of maltreatment as a risk factor for injury death: a prospective birth cohort study | (Putnam-Hornstein, 2011) | 596,962 | USA  (California) | <5 yrs | Physical, Psychological, Sexual | Health |
| 2011 | Understanding the mechanism behind maternal imprisonment and adolescent school dropout | (Cho, 2011) | 2,109 | USA  (Illinois) | 14 yrs | Criminal Behaviour | Education |
| 2012 | Children and Young People at Risk of Social Exclusion: Links between Homelessness, Child Protection and Juvenile Justice. Data Linkage Series. Number 13 | (Australian Institute of Health and Welfare, 2012) | Not stated | Australia  (Tasmania, Victoria) | 0-18 yrs  (Assumed) | Physical, Psychological, Sexual | Welfare, Justice |
| 2012 | Does Subsequent Criminal Justice Involvement Predict Foster Care and Termination of Parental Rights for Children Born to Incarcerated Women? | (Kubiak et al., 2012) | 83 | USA | Birth – 10 yrs | Criminal Behaviour, Substance Abuse | Welfare |
| 2012 | Early Morbidity and Mortality Following In Utero Exposure to Selective Serotonin Reuptake Inhibitors | (Colvin et al., 2012) | 3,764 | Australia  (Western Australia) | Prenatal | Mental Illness | Health |
| 2012 | Exposure to parental mortality and markers of morbidity, and the risks of attempted and completed suicide in offspring: an analysis of sensitive life periods | (Niederkrotenthaler, Floderus, et al., 2012) | 1,407 | Sweden | <3 yrs  3–10 yrs  >10 yrs | Mental Illness | Health |
| 2012 | Foster care re-entry: Exploring the role of foster care characteristics, in-home child welfare services and cross-sector services | (Lee et al., 2012) | 846 | USA  (Midwestern area) | <2 yrs  2-4 yrs  5-11 yrs  12+ yrs | Mental Illness, Substance Abuse, Criminal Behaviour, Psychological, Physical, Sexual | Welfare |
| 2012 | Health service use in families where children enter public care: a nested case control study using the General Practice Research Database | (Simkiss et al., 2012) | 2,954 | United Kingdom | Birth – 16 yrs | Mental Illness, Substance Abuse, Domestic Violence | Welfare |
| 2012 | Heavy maternal alcohol consumption and cerebral palsy in the offspring | (C. M. O'Leary et al., 2012) | 80,364 | Australia  (Western Australia) | During pregnancy,  ≤1 yr before pregnancy,  ≤1 yr after pregnancy,  >1 yr before pregnancy,  >1 yr after pregnancy | Substance Abuse | Health |
| 2012 | Heavy prenatal alcohol exposure and increased risk of stillbirth | (C. O'Leary et al., 2012) | 85,083 | Australia  (Western Australia) | During pregnancy,  ≤1 yr before pregnancy,  ≤1 yr after pregnancy,  >1 yr before pregnancy,  >1 yr after pregnancy | Substance Abuse | Health |
| 2012 | Out of care and into care again: A Danish register‐based study of children placed in out-of‐home care before their third birthday | (Ubbesen et al., 2012) | 3,928 | Denmark | <3 yrs | Mental Illness | Welfare |
| 2012 | Perinatal conditions and parental age at birth as risk markers for subsequent suicide attempt and suicide: a population based case-control study | (Niederkrotenthaler, Rasmussen, et al., 2012) | 18,566 | Sweden | 3-16 yrs | Mental Illness | Health |
| 2012 | Single Types of Family Violence Victimization and Externalizing Behaviors Among Children and Adolescents | (Renner, 2012) | 2,572 | USA  (Illinois) | 3-18 yrs | Physical, Psychological, Sexual | Health |
| 2012 | The interaction of parental history of suicidal behavior and exposure to adoptive parents' psychiatric disorders on adoptee suicide attempt hospitalizations | (Wilcox et al., 2012) | 8,391 | Sweden | <18 yrs | Mental Illness | Health |
| 2013 | Alcohol-use disorders during and within one year of pregnancy: a population-based cohort study 1985-2006 | (C. M. O'Leary et al., 2013) | 8,422 | Australia  (Western Australia) | During pregnancy, Within 1 year before pregnancy, Within 1 year after pregnancy | Substance Abuse | Health |
| 2013 | Child maltreatment and repeat presentations to the emergency department for suicide-related behaviors | (Rhodes et al., 2013) | 179 | Canada  (Ontario) | Birth – 16 yrs | Physical, Psychological, Sexual | Health |
| 2013 | Dental hospital admissions in the children of mothers with an alcohol-related diagnosis: a population-based, data-linkage study | (O'Leary & Slack-Smith, 2013) | 19,316 | Australia  (Western Australia) | <5 yrs | Substance Abuse | Health |
| 2013 | Exploring the potential to use data linkage for investigating the relationship between birth defects and prenatal alcohol exposure | (Colleen M. O'Leary et al., 2013) | 23,859 | Australia  (Western Australia) | Prenatal | Substance Abuse, Mental Illness | Health |
| 2013 | Intellectual disability: population-based estimates of the proportion attributable to maternal alcohol use disorder during pregnancy | (C. O'Leary et al., 2013) | 64,842 | Australia  (Western Australia) | Prenatal | Substance Abuse | Health |
| 2013 | Interaction between parental psychosis and risk factors during pregnancy and birth for schizophrenia – The Northern Finland 1966 Birth Cohort study | (Keskinen et al., 2013) | 10,526 | Finland | Prenatal | Mental Illness | Health |
| 2013 | Placement of children in out-of-home care in Québec, Canada: When and for whom initial out-of-home placement is most likely to occur | (Esposito et al., 2013) | 127,181 | Canada  (Quebec) | 0-1 yrs  2-5 yrs  6-9 yrs  14-17 yrs | Physical, Psychological, Sexual, Domestic Violence, Substance Abuse | Welfare |
| 2013 | Prenatal stress and affective disorders in a population birth cohort | (Kleinhaus et al., 2013) | 90,079 | Israel | Prenatal | Mental Illness | Health |
| 2013 | Reunification in a complete entry cohort: A longitudinal study of children entering out-of-home care in Tasmania, Australia | (Delfabbro et al., 2013) | 468 | Australia  (Tasmania) | 0-4 yrs  5-9 yrs  10-12 yrs  13 + | Physical, Psychological, Sexual, Substance Abuse, Criminal Behaviour, Mental Illness | Welfare |
| 2013 | Temporal stability of entries and predictors for entry into out-of-home care before the third birthday: A Danish population-based study of entries from 1981 to 2008 | (Ubbesen, Petersen, Mortensen, et al., 2013) | 11,034 | Denmark | <3 yrs | Mental Illness | Welfare |
| 2013 | The elementary-school functioning of children with maltreatment histories and mild cognitive or behavioral disabilities: A mixed methods inquiry | (Haight et al., 2013) | 10,394 | USA  (Minnesota) | School grades 3-6 | Physical, Psychological, Sexual | Education |
| 2013 | The transition from in-home services to out-of-home care: A Danish register-based study | (Ubbesen, Petersen, & Kristensen, 2013) | 8,232 | Denmark  (Aarhus) | 0-2 yrs  3-6 yrs  7-12 yrs  13-17 yrs | Mental Illness | Welfare |
| 2014 | A cross-sectional examination of birth rates among adolescent girls in foster care | (King et al., 2014) | 62,402 | USA  (California) | <17 yrs | Physical, Psychological, Sexual | Health |
| 2014 | An investigation of the relations between school concentrations of student risk factors and student educational well-being | (Fantuzzo et al., 2014) | 10,639 | USA  (Philadelphia) | <3^rd^ grade | Physical, Psychological, Sexual | Education |
| 2014 | Cumulative teen birth rates among girls in foster care at age 17: An analysis of linked birth and child protection records from California | (Putnam-Hornstein & King, 2014) | 20,222 | USA  (California) | <17 yrs | Physical, Psychological, Sexual | Health |
| 2014 | Maternal mental health and substance use disorders in sudden unexpected death in infancy using routinely collected health data in New Zealand, 2000-2016 | (Mitchell et al., 2022) | 1,079,811 | New Zealand | Prenatal | Mental Illness, Substance Abuse | Health |
| 2014 | Mental health at the intersections: the impact of complex needs on police contact and custody for Indigenous Australian men | (Trofimovs & Dowse, 2014) | 131 | Australia  (New South Wales) | Child | Physical, Psychological, Sexual | Justice |
| 2014 | Parental incarceration and child mortality in Denmark | (Wildeman et al., 2014) | 58,848 | Denmark | Child | Criminal Behaviour | Health |
| 2014 | Pregnancy, prison and perinatal outcomes in New South Wales, Australia: a retrospective cohort study using linked health data. | (Walker et al., 2014) | 302 | Australia  (New South Wales) | Birth–16 yrs  16-20 yrs | Criminal Behaviour, Substance Abuse, Mental Illness | Health |
| 2014 | The Effect of Paternal Incarceration on Children's Risk of Foster Care Placement | (Andersen & Wildeman, 2014) | 11,492 | Denmark | Child  Teenager | Criminal Behaviour | Welfare |
| 2014 | The influence of childhood sexual abuse on adolescent outcomes: the roles of gender, poverty, and revictimization | (Matta Oshima et al., 2014) | 555 | USA  (Midwest) | <11 yrs | Sexual | Health, Justice |
| 2015 | A population-level and longitudinal study of adolescent mothers and intergenerational maltreatment | (Putnam-Hornstein et al., 2015) | 85,084 | USA  (California) | 10-17 yrs | Physical, Psychological, Sexual | Welfare |
| 2015 | Child welfare services involvement among the children of young parents in foster care | (Dworsky, 2015) | 2,487 | USA  (Los Angeles County) | <19 yrs | Physical, Psychological, Sexual | Welfare |
| 2015 | Childhood residential mobility, schizophrenia, and bipolar disorder: a population-based study in Denmark | (Paksarian et al., 2015) | 1,100,000 | Denmark | 0-15 yrs | Mental Illness | Health |
| 2015 | Childhood adversity and behavioral health outcomes for youth: An investigation using state administrative data | (Lucenko et al., 2015) | 125,123 | USA  (Washington) | <17 yrs | Domestic Violence, Mental Illness, Criminal behaviour, Physical, Psychological, Sexual | Health |
| 2015 | Infant and maternal characteristics in neonatal abstinence syndrome--selected hospitals in Florida, 2010-2011 | (Lind et al., 2015) | 242 | USA  (Florida | Prenatal | Substance Abuse | Health |
| 2015 | Intergenerational effects of parental substance-related convictions and adult drug treatment court participation on children's school performance | (Gifford et al., 2015) | 845,613 | USA  (North Carolina) | <8^th^ grade | Substance Abuse | Education |
| 2015 | Maternal mental health and risk of child protection involvement: mental health diagnoses associated with increased risk | (O'Donnell et al., 2015) | 74,888 | Australia  (Western Australia) | Perinatal | Mental Illness | Welfare |
| 2015 | Parental mental illness and eating disorders in offspring | (Bould et al., 2015) | 158,679 | Sweden  (Stockholm) | 0-17 yrs | Mental Illness | Health |
| 2015 | Predictors of Childhood Anxiety: A Population-Based Cohort Study | (Kingston et al., 2015) | 19,316 | Canada  (Manitoba) | Birth–12 mo  13 mo–5 yrs | Mental Illness | Health |
| 2015 | Pregnancy outcomes of mothers with an alcohol-related diagnosis: a population-based cohort study for the period 1983-2007 | (Srikartika & O'Leary, 2015) | 23,573 | Australia  (Western Australia) | Infant – before, during, after pregnancy | Substance Abuse | Health |
| 2015 | The association between childhood maltreatment experiences and the onset of maltreatment perpetration in young adulthood controlling for proximal and distal risk factors | (Ben-David et al., 2015) | 6,935 | USA  (Midwestern metropolitan area) | <18 yrs | Physical, Psychological, Sexual | Welfare |
| 2015 | Triparental families: a new genetic-epidemiological design applied to drug abuse, alcohol use disorders, and criminal behavior in a Swedish national sample | (Kendler et al., 2015) | 41,360 | Sweden | 0-15 yrs | Criminal Behaviour, Substance Abuse | Health, Justice |
| 2015 | Understanding Overrepresentation of Indigenous Children in Child Welfare Data: An Application of the Drake Risk and Bias Models | (Cram et al., 2015) | 180,794 | New Zealand | 5 yrs before child’s birth | Criminal Behaviour | Health |
| 2016 | Child Welfare and the Transition to Adulthood: Investigating Placement Status and Subsequent Arrests | (Ryan et al., 2016) | 54,175 | USA  (Washington State) | <17 yrs | Physical, Psychological, Sexual | Justice |
| 2016 | Criminally Involved Parents Who Misuse Substances and Children's Odds of Being Arrested as a Young Adult: Do Drug Treatment Courts Mitigate the Risk? | (Gifford, Eldred, Evans, et al., 2016) | 29,903 | USA  (North Carolina) | 15-18 yrs | Criminal Behaviour, Substance Abuse | Justice |
| 2016 | Development of a prediction model for child maltreatment recurrence in Japan: A historical cohort study using data from a Child Guidance Center | (Horikawa et al., 2016) | 715 | Japan | 0-4 yrs  5-8 yrs  9-13 yrs  14-17 yrs | Substance Abuse, Domestic Violence, Mental Illness, Physical, Psychological, Sexual | Welfare |
| 2016 | Does poor school performance cause later psychosocial problems among children in foster care? Evidence from national longitudinal registry data | (Forsman et al., 2016) | 7,522 | Sweden | <13 yrs | Physical, Psychological, Sexual, Substance Abuse, Mental Illness, Criminal Behaviour | Health |
| 2016 | Entering out-of-home care during childhood: Cumulative incidence study in Canada and Australia | (O'Donnell et al., 2016) | 460,886 | Australia  (Western Australia)  Canada  (Manitoba) | Child | Domestic violence, Mental Illness, Substance Abuse, Criminal Behaviour | Welfare |
| 2016 | From maltreatment to delinquency: Service trajectories after a first intervention of child protection services | (Laurier et al., 2016) | 14,252 | Canada  (Quebec) | 0-5 yrs  6-11 yrs  12-17 yrs | Physical, Psychological, Sexual | Justice |
| 2016 | Parental alcohol-related disorders and school performance in 16-year-olds-a Swedish national cohort study | (Berg et al., 2016) | 740,618 | Sweden | Birth–15 yrs | Substance Abuse, Mental Illness | Education |
| 2016 | Parental Criminal Justice Involvement and Children's Involvement With Child Protective Services: Do Adult Drug Treatment Courts Prevent Child Maltreatment | (Gifford, Eldred, Sloan, et al., 2016) | 297,969 | USA  (North Carolina) | <1 yr  1-3 yrs  4-6 yrs  7-18 yrs | Substance Abuse, Criminal Behaviour | Welfare |
| 2016 | Parental death in childhood and self-inflicted injuries in young adults-a national cohort study from Sweden | (Rostila et al., 2016) | 871,402 | Sweden | 0-5 yrs  6-11 yrs  12-17 yrs | Mental Illness, Substance Abuse, Domestic Violence | Health |
| 2016 | Parental Psychiatric Disease and Risks of Attempted Suicide and Violent Criminal Offending in Offspring A Population-Based Cohort Study | (Mok et al., 2016) | 1,743,525 | Denmark | ≥15 yrs | Mental Illness, Substance Abuse | Health, Justice |
| 2016 | Pre-existing adversity, level of child protection involvement, and school attendance predict educational outcomes in a longitudinal study | (Maclean et al., 2016) | 46,838 | Australia  (Western Australia) | Birth – Yr 3 (school) | Physical, Psychological, Sexual, Mental Illness, Substance Abuse, Criminal Behaviour | Education |
| 2016 | Preventing intellectual disability during pregnancy: what are the potentially high yield targets? | (Wang et al., 2016) | 123,922 | USA  (South Carolina) | Prenatal | Mental Illness | Health |
| 2016 | Psychosocial characteristics and predictors of health-care use in families of young children with cystic fibrosis in Western Australia | (Douglas et al., 2016) | 75 | Australia  (Western Australia) | 0-1 yrs  2-3 yrs  4-5 yrs | Mental Illness | Health |
| 2016 | Sexually Transmitted Infections In A Sample Of At-Risk Youth: Roles Of Mental Health And Trauma Histories | (Gerassi et al., 2016) | 4,117 | USA  (Midwest) | Birth – 11 yrs | Physical, Psychological, Sexual, Substance Abuse | Health |
| 2016 | Social, familial and psychological risk factors for psychosis: A birth cohort study using the Danish Registry System | (Shevlin et al., 2016) | 54,458 | Denmark | <10 yrs | Mental Illness, Physical Psychological, Sexual | Health |
| 2016 | The relationship between child maltreatment, intimate partner violence exposure, and academic performance | (Kiesel et al., 2016) | 2,914 | USA  (Minnesota) | 7-13 yrs | Physical, Psychological, Sexual, Domestic Violence | Education |
| 2016 | The variation in family background amongst young homeless shelter users in Denmark | (Benjaminsen, 2016) | About 54,000 | Denmark | Childhood | Physical, Psychological, Sexual, Mental Illness, Substance Abuse | Health |
| 2016 | What predictors matter: Risk factors for late adolescent outcomes | (Wall-Wieler et al., 2016) | 92,183 | Canada  (Manitoba) | 9-13 yrs | Physical, Psychological, Sexual | Education, Health |
| 2017 | A Longitudinal Study of Child Maltreatment and Mental Health Predictors of Admission to Psychiatric Residential Treatment Facilities | (Rose & Lanier, 2017) | 105,982 | USA  (North Carolina) | <17 yrs | Physical, Psychological, Sexual | Health |
| 2017 | Academic Performance in Children of Mothers With Schizophrenia and Other Severe Mental Illness, and Risk for Subsequent Development of Psychosis: A Population-Based Study | (Lin et al., 2017) | 15,486 | Australia  (Western Australia) | Birth – 5 yrs | Mental Illness | Education |
| 2017 | Adverse childhood or adult experiences and risk of bilateral oophorectomy: a population-based case-control study | (Gazzuola Rocca et al., 2017) | 128 | USA (Minnesota) | Birth – 19 yrs | Mental Illness, Substance Abuse, Domestic Violence, Physical, Psychological, Sexual | Health |
| 2017 | Association Between Serotonergic Antidepressant Use During Pregnancy and Autism Spectrum Disorder in Children | (Brown et al., 2017) | 35,906 | Canada  (Ontario) | Prenatal | Mental Illness | Health |
| 2017 | Childhood maltreatment and high dietary fat intake behaviors in adulthood: A birth cohort study | (Abajobir et al., 2017) | 3,766 | Australia  (Queensland) | 0-14 yrs | Physical, Psychological, Sexual | Health |
| 2017 | Cumulative risk over the early life course and its relation to academic achievement in childhood and early adolescence | (Ragnarsdottir et al., 2017) | 1,151 | Iceland  (Reykjavik) | 0-13 yrs | Physical, Psychological, Sexual | Education |
| 2017 | Effects of maltreatment and parental schizophrenia spectrum disorders on early childhood social-emotional functioning: a population record linkage study | (Matheson et al., 2017) | 69,116 | Australia  (New South Wales) | Birth – 5 yrs | Mental Illness, Physical, Psychological, Sexual | Health |
| 2017 | Factors associated with re-entry to out-of-home care among children in England | (Mc Grath-Lone et al., 2017) | 95,369 | England | <16 yrs | Physical, Psychological, Sexual | Welfare |
| 2017 | First Births to Maltreatment Adolescent Girls: Differences Associated With Spending Time in Foster Care | (King, 2017) | 85,766 | USA  (California) | > 10 yrs | Physical, Psychological, Sexual | Health |
| 2017 | Further victimization of child sexual abuse victims: A latent class typology of re-victimization trajectories | (Papalia et al., 2017) | 401 | Australia  (Victoria) | <12 yrs  12-16 yrs | Sexual | Health, Justice |
| 2017 | Impact of social disadvantage and parental offending on rates of criminal offending among offspring of women with severe mental illness | (Valuri et al., 2017) | 467,945 | Australia  (Western Australia) | Child | Mental Illness, Criminal Behaviour | Justice |
| 2017 | Influence of exposure to perinatal risk factors and parental mental health related hospital admission on adolescent deliberate self-harm risk | (Hu, Li, et al., 2017) | 474,860 | Australia  (Western Australia) | <19 yrs | Mental Illness | Health |
| 2017 | Maternal alcohol disorders and school achievement: a population cohort record linkage study in Western Australia | (Johnson et al., 2017) | 66,748 | Australia  (Western Australia) | >1 yr pre pregnancy,  Up to 1 yr pre pregnancy,  During pregnancy,  Up to 1 yr post pregnancy,  >1 yr post pregnancy | Substance Abuse | Education |
| 2017 | Maternal alcohol use disorder and child school attendance outcomes for non-Indigenous and Indigenous children in Western Australia: a population cohort record linkage study | (K. Hafekost et al., 2017) | 253,714 | Australia  (Western Australia) | During pregnancy,  >1 yr post pregnancy,  >1 yr pre pregnancy,  Up to 1 yr post pregnancy,  Up to 1 yr pre pregnancy | Substance Abuse | Education |
| 2017 | Maternal Alcohol Use Disorder and Risk of Child Contact with the Justice System in Western Australia: A Population Cohort Record Linkage Study | (Katherine Hafekost, David Lawrence, Colleen O'Leary, et al., 2017) | 253,714 | Australia  (Western Australia) | Child | Substance Abuse, Mental Illness | Justice, Education |
| 2017 | Maternal alcohol use disorder and subsequent child protection contact: A record-linkage population cohort study | (Katherine Hafekost, David Lawrence, Colleen O’Leary, et al., 2017) | 6,568 | Australia  (Western Australia) | During pregnancy,  >1 yr post pregnancy,  >1 yr pre-pregnancy,  Up to 1 yr post pregnancy,  Up to 1 yr pre-pregnancy | Substance Abuse | Welfare |
| 2017 | Maternal depression and anxiety disorders (MDAD) and child development: A Manitoba population-based study | (Comaskey et al., 2017) | 18,331 | Canada  (Manitoba) | One year prior to child’s birth to Kindergarten year | Mental Illness | Health, Education |
| 2017 | Maternal Mental Health Disorders and Reports to Child Protective Services: A Birth Cohort Study | (Hammond et al., 2017) | 551,232 | USA  (California) | Prenatal | Mental Illness | Welfare |
| 2017 | Parental Psychopathology and Tourette Syndrome/Chronic Tic Disorder in Offspring: A Nationwide Case-Control Study | (Leivonen et al., 2017) | 5,419 | Finland | Child | Mental Illness, Substance Abuse | Health |
| 2017 | Parental substance use and foster care reentry | (Mowbray et al., 2017) | 17,420 | USA  (Midwest) | Youth | Substance Abuse, Domestic Violence, Physical, Psychological, Sexual | Welfare |
| 2017 | Pervasive influence of maternal and paternal criminal offending on early childhood development: a population data linkage study | (Laurens et al., 2017) | 66,477 | Australia  (New South Wales) | <5 yrs | Criminal Behaviour | Health, Education |
| 2017 | Post-traumatic Stress Disorder and Antepartum Complications: a Novel Risk Factor for Gestational Diabetes and Preeclampsia | (Shaw et al., 2017) | 15,986 | USA | Prenatal | Mental Illness | Health |
| 2017 | Relationship between out-of-home care placement history characteristics and educational achievement: A population level linked data study | (Maclean et al., 2017) | 235,045 | Australia  (Western Australia) | <Yr 3 (school) | Physical, Psychological, Sexual, Mental Illness | Education |
| 2017 | Risk factors for fatal and non-fatal child maltreatment in families previously investigated by CPS: A case-control study | (Miyamoto et al., 2017) | 702 | USA  (Northern California) | <6 yrs | Substance Abuse, Domestic Violence, Mental Illness, Criminal Behaviour | Welfare, Health |
| 2017 | The Hidden Cost of Foster-Care: New Evidence on the Inter-Generational Transmission of Foster-Care Experiences | (Mertz & Andersen, 2017) | 15,213 | Denmark | 0-18 yrs | Physical, Psychological, Sexual | Welfare |
| 2017 | The impact of child maltreatment on the risk of deliberate self-harm among adolescents: A population-wide cohort study using linked administrative records | (Hu, Taylor, et al., 2017) | 351,372 | Australia  (Western Australia) | 0-9 yrs  10-19 yrs  Or both | Physical, Psychological, Sexual | Health |
| 2017 | The impact of parental offending on offspring aggression in early childhood: a population-based record linkage study | (Tzoumakis et al., 2017) | 69,116 | Australia  (New South Wales) | <5 yrs | Criminal Behaviour, Mental Illness | Health |
| 2017 | The placement trajectories of youth served by child protection for sexual abuse | (Esposito et al., 2017) | 77,579 | Canada (Quebec) | 10-17 yrs | Physical, Psychological, Sexual | Welfare |
| 2017 | Transitions and turning points revisited: A replication to explore child maltreatment and youth offending links within and across Australian cohorts | (Hurren et al., 2017) | 4,511 | Australia  (Queensland) | Birth – 18 yrs | Physical, Psychological, Sexual | Justice |
| 2018 | Age-specific risk factors associated with placement instability among foster children | (Sattler et al., 2018) | 26,113 | USA  (Texas) | 0-5 yrs  6-12 yrs  13 + yrs | Physical, Psychological, Sexual | Welfare |
| 2018 | Assortative mating and the intergenerational transmission of parental incarceration risks | (Andersen, 2018) | 256,931 | Denmark | <15 yrs | Criminal Behaviour | Justice |
| 2018 | Chains of risk for alcohol use disorder: Mediators of exposure to neighborhood deprivation in early and middle childhood | (Karriker-Jaffe et al., 2018) | 883,969 | Sweden | Early and middle childhood | Substance Abuse, Criminal Behaviour | Health |
| 2018 | Characteristics of foster care history as risk factors for psychiatric disorders among youth in care | (Okpych & Courtney, 2018) | 763 | USA  (California) | Child/Youth | Physical, Psychological, Sexual | Health |
| 2018 | Child maltreatment and mental health problems in adulthood: birth cohort study | (Kisely et al., 2018) | 3,778 | Australia  (Brisbane) | <16 yrs | Physical, Psychological, Sexual | Health |
| 2018 | Childhood developmental vulnerabilities associated with early life exposure to infectious and noninfectious diseases and maternal mental illness | (Green, Kariuki, et al., 2018) | 66,045 | Australia  (New South Wales) | Before or after childbirth | Mental Illness | Health, Education |
| 2018 | Childhood Maltreatment and Early Developmental Vulnerabilities at Age 5 Years | (Green, Tzoumakis, McIntyre, et al., 2018) | 64,459 | Australia  (New South Wales) | <19 mos  19-36 mo  ≥37 mo | Physical, Psychological, Sexual | Health, Education |
| 2018 | Determinants of infant mortality for children of women prisoners: A longitudinal linked data study | (C. M. M. Dowell et al., 2018) | 42,674 | Australia  (Western Australia) | During pregnancy, before (but not during) pregnancy, after birth | Criminal Behaviour, Mental Illness, Substance Abuse | Health |
| 2018 | Excess mortality in children born to opioid-addicted parents: A national register study in Taiwan | (Fang et al., 2018) | 3,210 | Taiwan | <6 yrs | Substance Abuse, Mental Illness | Health |
| 2018 | Factors associated with child protection recurrence in Australia | (Jenkins et al., 2018) | 9,608 | Australia  (Queensland) | Child | Physical, Psychological, Sexual, Mental Illness, Criminal Behaviour, Substance Abuse | Welfare |
| 2018 | Gastro-oesophageal reflux: a mixed methods study of infants admitted to hospital in the first 12 months following birth in NSW (2000-2011) | (Dahlen et al., 2018) | 11,513 | Australia  (New South Wales) | 12 mo after birth | Mental Illness | Health |
| 2018 | Human trafficking and the child welfare population in Florida | (Gibbs et al., 2018) | 3,420 | USA  (Florida) | >10 yrs | Physical, Psychological, Sexual | Welfare |
| 2018 | Intellectual Disability and Psychotic Disorders in Children: Association With Maternal Severe Mental Illness and Exposure to Obstetric Complications in a Whole-Population Cohort | (Di Prinzio et al., 2018) | 15,351 | Australia  (Western Australia) | Child | Mental Illness | Health |
| 2018 | Latent profiles of early developmental vulnerabilities in a New South Wales child population at age 5 years | (Green, Tzoumakis, Laurens, et al., 2018) | 67,353 | Australia  (New South Wales) | 3 yrs prior to birth – 5 yrs | Physical, Psychological, Sexual, Substance Abuse, Mental Illness, Criminal Behaviour | Health, Education |
| 2018 | Linking Healthcare and Social Service Databases to Study the Epidemiology of Child Maltreatment  and Associated Health Problems: Hong Kong's Experience | (Lo et al., 2018) | 4,816 | Hong Kong | <18 yrs | Physical, Psychological, Sexual | Health |
| 2018 | Maternal incarceration, child protection, and infant mortality: a descriptive study of infant children of women prisoners in Western Australia | (C. M. Dowell et al., 2018) | 349 | Australia  (Western Australia) | Birth – 2 yrs | Criminal Behaviour | Welfare, Health |
| 2018 | Out-of-Home Care and the Educational Achievement, Attendance, and Suspensions of Maltreated Children: A Propensity-Matched Study | (Maclean et al., 2018) | 3,297 | Australia  (Western Australia) | <Yr 9 (school) | Physical, Psychological, Sexual, Mental Illness, Substance Abuse, Domestic Violence | Education |
| 2018 | Out-of-home placement in early childhood and psychiatric diagnoses and criminal convictions in young adulthood:  a population-based propensity score-matched study | (Côté et al., 2018) | 59,476 | Finland | 2-6 yrs | Physical, Psychological, Sexual | Health, Justice |
| 2018 | Post-Traumatic Hydrocephalus in Children: A Retrospective Study in 42 Pediatric Hospitals Using the Pediatric Health Information System | (Bonow et al., 2018) | 91,583 | USA | <1 yr  1-5 yrs  6-11 yrs  12-21 yrs | Physical | Health |
| 2018 | Predicting chronic neglect: Understanding risk and protective factors for CPS-involved families | (Logan‐Greene & Semanchin Jones, 2018) | 2,074 | USA  (Northeast city) | Child | Domestic Violence, Substance Abuse, Mental Illness | Welfare |
| 2018 | Predictors of having a first child taken into care at birth: A population-based retrospective cohort study | (Wall-Wieler et al., 2018) | 53,565 | Canada (Manitoba) | Two years before pregnancy - pregnancy | Substance Abuse, Mental Illness, Criminal Behaviour | Welfare |
| 2018 | Prenatal maternal smoking, maternal offending, and offspring behavioural and cognitive outcomes in early childhood | (Tzoumakis et al., 2018) | 66,523 | Australia  (New South Wales) | <5 yrs | Criminal Behaviour, Substance Abuse, Mental Illness | Health, Education |
| 2018 | Report from CalYOUTH: Findings on the Relationship between Extended Foster Care and Youth’s Outcomes at Age 21 | (Courtney et al., 2018) | Over 40,000 | USA  (California) | 16-21 yrs | Physical, Psychological, Sexual | Education, Health, Justice |
| 2018 | Risk of serious criminal involvement among former foster youth aging out of care | (Crawford et al., 2018) | 1,420 | USA  (Southwestern state) | Child | Physical, Psychological, Sexual, Substance Abuse, Criminal Behaviour | Justice |
| 2018 | The impact of parental mental illness across the full diagnostic spectrum on externalising and internalising vulnerabilities in young offspring | (Dean et al., 2018) | 69,116 | Australia  (New South Wales) | <5 yrs | Mental Illness | Health, Education |
| 2018 | Using State Administrative Data to Identify Social Complexity Risk Factors for Children | (Arthur et al., 2018) | 505,367 | USA  (Washington State) | <5 yrs  5-17 yrs | Physical, Psychological, Sexual, Substance Abuse, Mental Illness, Criminal Behaviour, Domestic Violence | Health |
| 2018 | Using linked data to investigate developmental vulnerabilities in children of convicted parents | (Bell et al., 2018) | 19,071 | Australia  (Western Australia) | 12 months prior to birth – 5 yrs | Criminal Behaviour | Health, Education |
| 2019 | A Descriptive Analysis of the Child Protection Histories of Youth and Young Adults Arrested in California | (Eastman et al., 2019) | 394,377 | USA  (California) | Child | Physical, Psychological, Sexual | Justice |
| 2019 | A large proportion of poor birth outcomes among Aboriginal Western Australians are attributable to smoking, alcohol and substance misuse, and assault | (Gibberd et al., 2019) | 28,119 | Australia  (Western Australia) | 2 yrs prior to birth - birth | Substance Abuse, Domestic Violence | Health |
| 2019 | A prospective, longitudinal study of risk factors for early onset of delinquency among maltreated youth | (Cho et al., 2019) | 5,002 | USA  (Minnesota) | <14 yrs | Physical, Psychological, Sexual | Justice |
| 2019 | Are familial liability for schizophrenia and obstetric complications independently associated with risk of a psychotic illness, after adjusting for other environmental stressors in childhood? | (Morgan et al., 2019) | 298,370 | Australia (Western Australia) | Up to 18 yrs | Mental Illness, Physical, Psychological, Sexual | Health |
| 2019 | Assessing the social determinants of health care costs for Medicaid-enrolled adolescents in Washington State using administrative data | (Patton et al., 2019) | 233,054 | USA (Washington State) | 0-3 yrs  4-11 yrs  12+ yrs | Physical, Psychological, Sexual, Criminal Behaviour, Mental Illness | Health |
| 2019 | Association of Childhood Exposure to Nitrogen Dioxide and Polygenic Risk Score for Schizophrenia With the Risk of Developing Schizophrenia | (Horsdal et al., 2019) | 23,355 | Denmark | Birth – 10 yrs | Mental Illness | Health |
| 2019 | Children of parents who have been hospitalised with psychiatric disorders are at risk of poor school readiness | (Bell et al., 2019) | 19,071 | Australia  (Western Australia) | 12 mos prior to birth – 5 yrs | Mental Illness | Health, Education |
| 2019 | Early developmental risk for subsequent childhood mental disorders in an Australian population cohort | (Green, Tzoumakis, et al., 2019) | 86,668 | Australia  (New South Wales) | 3 years prior to birth – 13 yrs | Physical, Psychological, Sexual, Mental Illness | Health |
| 2019 | Early life adversity, contact with children's social care services and educational outcomes at age 16 years: UK birth cohort study with linkage to national administrative records | (Teyhan et al., 2019) | 12,868 | United Kingdom  (Bristol) | 1-18 yrs | Physical, Psychological, Sexual | Education |
| 2019 | Effects of Parental Opioid Use: Outcomes of Children of Parents in Medication-Assisted Treatment Compared to Healthy Controls | (Betcher et al., 2019) | 144 | USA  (Minnesota) | Prenatal | Substance Abuse | Health, Welfare |
| 2019 | Elevated maternal and child mortality among women with multiple DUI convictions compared with socio-demographically matched controls | (McCutcheon et al., 2019) | 1,658 | USA  (Missouri) | Prenatal | Substance Abuse | Health |
| 2019 | Foster care outcomes and experiences of infants removed due to substance abuse | (Boyd, 2019) | 32,117 | USA | <1 yr | Substance Abuse | Welfare |
| 2019 | From population reference to national standard: new and improved birthweight charts | (Hoftiezer et al., 2019) | 2,712,301 | The Netherlands | Prenatal | Substance Abuse | Health |
| 2019 | Hearing and justice: The link between hearing impairment in early childhood and youth offending in Aboriginal children living in remote communities of the Northern Territory, Australia | (He et al., 2019) | 1,533 | Australia (Northern Territory) | <Yr 7 (school) | Physical, Psychological, Sexual | Justice |
| 2019 | Hospitalisations for maternal assault are associated with increased risk of child protection involvement | (Orr et al., 2019) | 524,534 | Australia  (Western Australia) | 12 months prior to birth, 12 months prior to birth and at any point in child’s life | Domestic Violence | Welfare |
| 2019 | Human trafficking victimization among youth who run away from foster care | (Latzman et al., 2019) | 36,997 | USA  (Florida) | Birth – 18 yrs | Physical, Psychological, Sexual | Welfare |
| 2019 | Inter-agency indicators of out-of-home-care placement by age 13–14 years: A population record linkage study | (Green, Kariuki, et al., 2019) | 72,079 | Australia  (New South Wales) | Child | Mental Illness, Criminal Behaviour | Welfare |
| 2019 | Low birth weight and maternal incarceration in pregnancy: A longitudinal linked data study of Western Australian infants | (Dowell et al., 2019) | 41,910 | Australia  (Western Australia) | Prenatal | Criminal Behaviour, Substance Abuse, Mental Illness | Health |
| 2019 | Modelling factors for Aboriginal and Torres Strait Islander child neurodevelopment outcomes: A latent class analysis | (Strobel et al., 2019) | 2,715 | Australia  (Western Australia) | 1 yr before birth – 5 yrs | Physical, Psychological, Sexual, Mental Illness | Health, Education |
| 2019 | Overcrowded housing: One of a constellation of vulnerabilities for child sexual abuse | (Cant et al., 2019) | 524,478 | Australia  (Western Australia) | Child | Mental Illness, Substance Abuse | Welfare |
| 2019 | Parental alcohol use disorder and offspring marital outcome | (Salvatore et al., 2019) | 1,171,070 | Sweden | Child | Substance Abuse, Criminal Behaviour | Health |
| 2019 | Parental offending and children's emergency department presentations in New South Wales, Australia | (Tyson Whitten et al., 2019) | 72,772 | Australia  (New South Wales) | 2-12 yrs | Criminal Behaviour | Health |
| 2019 | Prevalence and Risk Factors for Early Motherhood Among Low-Income, Maltreated, and Foster Youth | (Font et al., 2019) | 71,824 | USA (Wisconsin) | <18 yrs | Physical, Psychological, Sexual | Health |
| 2019 | Role of pre-existing adversity and child maltreatment on mental health outcomes for children involved in child protection: population-based data linkage study | (Maclean et al., 2019) | 524,534 | Australia (Western Australia) | Child | Physical, Psychological, Sexual | Health |
| 2019 | Social, familial and psychological risk factors for endocrine, nutritional and metabolic disorders in childhood and early adulthood: A birth cohort study using the Danish registry system | (Shevlin et al., 2019) | 54,458 | Denmark | Child | Physical, Psychological, Sexual | Health |
| 2019 | The influence of parental offending on the continuity and discontinuity of children's internalizing and externalizing difficulties from early to middle childhood | (T. Whitten et al., 2019) | 19,208 | Australia  (New South Wales) | Prior to birth,  Early childhood,  Middle childhood,  Both early and middle childhood | Criminal Behaviour | Health, Education |
| 2019 | Timing of the first report and highest level of child protection response in association with early developmental vulnerabilities in an Australian population cohort | (Rossen et al., 2019) | 10,944 | Australia  (New South Wales) | 0-18 mos (incl pre-birth),  19-36 mos  ≥37 mos | Physical, Psychological, Sexual | Education, Health |
| 2019 | Trajectory of service use among Albertan youth with complex service need | (Izakian et al., 2019) | 49,904 | Canada  (Alberta) | <15 yrs | Physical, Psychological, Sexual | Health, Education |
| 2020 | Adolescent education outcomes and maltreatment: The role of pre-existing adversity, level of child protection involvement, and school attendance | (Maclean et al., 2020) | 33,866 | Australia  (Western Australia) | <Yr 3 (school) | Physical, Psychological, Sexual, Mental Illness, Substance Abuse | Education |
| 2020 | Adverse childhood experiences during childhood and academic attainment at age 7 and 11 years: an electronic birth cohort study | (Evans et al., 2020) | 151,127 | United Kingdom  (Wales) | Birth- 6-7 yrs  Birth 10-11 yrs | Mental Illness, Substance Abuse | Education |
| 2020 | Child maltreatment and mental health problems in 30-year-old adults: A birth cohort study | (Kisely, Strathearn, et al., 2020) | 2,861 | Australia  (Queensland) | <16.5 yrs | Physical, Psychological, Sexual | Health |
| 2020 | Child Maltreatment and Persistent Smoking From Adolescence Into Adulthood: A Birth Cohort Study | (Kisely, Abajobir, et al., 2020) | 3,758 | Australia  (Brisbane) | <16 yrs | Physical, Psychological, Sexual | Health |
| 2020 | Child maltreatment and psychiatric outcomes in early adulthood | (Murphy et al., 2020) | 4,718 | Denmark | Child | Substance Abuse | Health |
| 2020 | Child, Family, and Case Characteristics Associated With Reentry Into Out-of-Home Care Among Children and Youth Involved With Child Protection Services | (Brown et al., 2020) | 16,814 | USA  (Colorado) | 0-4 yrs  5-11 yrs  12-16 yrs | Physical, Psychological, Sexual, Mental Illness, Substance Abuse | Welfare |
| 2020 | Does child maltreatment predict alcohol use disorders in young adulthood? A cohort study of linked notifications and survey data | (Kisely, Mills, et al., 2020) | 169 | Australia  (Queensland) | <14 yrs | Physical, Psychological, Sexual | Health |
| 2020 | Exposure to family and domestic violence is associated with increased childhood hospitalisations | (C. Orr et al., 2023) | 15,598 | Australia  (Western Australia) | 12 mos prior to birth – 5 yrs | Domestic Violence | Health |
| 2020 | Factors Associated with Child-Welfare Involvement among Prenatally Substance-Exposed Infants | (Deutsch et al., 2020) | 1,222 | USA  (Delaware) | Prenatal | Substance Abuse, Mental Illness, Physical, Psychological, Sexual | Welfare |
| 2020 | Hospitalizations among children involved in the child protection system: A long-term birth cohort study from infancy to adulthood using administrative data | (Gnanamanickam et al., 2020) | 608,540 | Australia  (South Australia) | Birth-adult | Physical, Psychological, Sexual | Health |
| 2020 | Impact of substance use and other risk factor exposures on conviction rates by people with a psychotic illness and other mental disorders | (Valuri et al., 2020) | 495,945 | Australia  (Western Australia) | Child | Physical, Psychological, Sexual, Criminal Behaviour, Mental Illness | Justice |
| 2020 | Incidence and Risk Factors for Abusive Head Trauma: A Population-Based Study | (Rebbe et al., 2020) | 354 | USA  (Washington State) | <5 yrs | Physical, Psychological, Sexual | Health |
| 2020 | Matched cohort study of healthcare resource utilization and costs in young children of mothers with postpartum depression in the United States | (Moore Simas et al., 2020) | 135,678 | USA | 15-365 days after delivery | Mental Illness | Health |
| 2020 | Maternal Alcohol-Use Disorder and Child Outcomes | (O'Leary et al., 2020) | 14,932 | Australia  (Western Australia) | During pregnancy,  ≤1 yr before pregnancy,  >1 yr before pregnancy or after pregnancy  ≤1 year after pregnancy  >1 yr before pregnancy | Substance Abuse | Health, Education, Justice, Welfare |
| 2020 | Maternal and child delivery charges, postpartum charges, and utilization of care among women with bipolar disorder: a cohort study | (Hoffman et al., 2020) | 4,440 | USA  (Denver) | <2 yrs | Mental Illness | Health |
| 2020 | Mental disorders in children known to child protection services during early childhood | (Green et al., 2020) | 74,462 | Australia  (New South Wales) | <6 yrs | Physical, Psychological, Sexual | Health |
| 2020 | Outcomes in Adulthood After Long-Term Foster Care: A Sibling Approach | (Brännström et al., 2020) | 4,116 | Sweden | <13 yrs | Physical, Psychological, Sexual | Education, Health, Justice |
| 2020 | Parsing components of risk of premature mortality in the children of mothers with severe mental illness | (Di Prinzio et al., 2020) | 15,486 | Australia  (Western Australia) | <5 yrs | Mental Illness | Health |
| 2020 | Predictors of remaining in foster care after age 18 years old | (Park et al., 2020) | 38,538 | USA (California) | <18 yrs | Physical, Psychological, Sexual | Welfare |
| 2020 | Reading and numeracy attainment of children reported to child protection services: A population record linkage study controlling for other adversities | (Laurens et al., 2020) | 56,860 | Australia  (New South Wales) | Birth – 8 yrs  Birth – 10 yrs | Physical, Psychological, Sexual, Mental Illness, Criminal Behaviour | Education |
| 2020 | Relationship between Child Sexual Abuse, Psychiatric Disorders and Infectious Diseases: A Matched-Cohort Study | (Maalouf et al., 2020) | 882 | Canada | <18 yrs | Sexual | Health |
| 2020 | Residential instability, running away, and juvenile detention characterizes commercially sexually exploited youth involved in Washington State's child welfare system | (Pullmann et al., 2020) | 83 | USA  (Washington State) | <14 yrs | Sexual | Welfare, Justice |
| 2020 | Risk of psychiatric diagnoses in children and adolescents of parents with torture trauma and war trauma | (Bager et al., 2020) | 19,294 | Denmark | Birth – 18 yrs | Mental Illness | Health |
| 2020 | School Absenteeism Associated With Child Protection System Involvement, Maltreatment Type, and Time in Out-of-Home Care | (Armfield et al., 2020) | 296,422 | Australia  (South Australia) | Child | Physical, Psychological, Sexual | Education |
| 2020 | Sleep Patterns, Problems and Ecology among Toddlers in Families with a Child Protective Services Maltreatment Referral | (Hash et al., 2020) | 113 | USA  (Washington State) | 10-24 mos | Physical, Psychological, Sexual | Health |
| 2020 | Somatic and mental symptoms, medical treatments and service use in aftercare-Document analysis of Finnish care leavers | (Toivonen et al., 2020) | 600 | Finland | <15.5-17 yrs  18-24 yrs | Physical, Psychological, Sexual, Substance Abuse, Mental Illness, Criminal Behaviour | Health |
| 2020 | The Intergenerational Impact of Terror: Did the 9/11 Tragedy Impact the Initial Human Capital of the Next Generation? | (Brown, 2020) | 27,568,056 | USA | Prenatal | Mental Illness, Substance Abuse | Health |
| 2020 | Transitions between socio-emotional and cognitive vulnerability profiles from early to middle childhood: a population study using multi-agency administrative records | (Piotrowska et al., 2020) | 19,087 | Australia  (New South Wales) | 0-5 yrs  6-11 yrs | Physical, Psychological, Sexual, Mental Illness, Criminal Behaviour | Health, Education |
| 2021 | "Who maltreats? Distinct pathways of intergenerational (dis)continuity of child maltreatment" | (McKenzie et al., 2021) | 32,574 | Australia  (Queensland) | 0-17 yrs | Physical, Psychological, Sexual | Welfare |
| 2021 | A comparison of trajectories of offending among people with psychotic disorders, other mental disorders and no mental disorders: Evidence from a whole-of-population birth cohort study | (Valuri et al., 2021) | 467,945 | Australia  (Western Australia) | <18 yrs | Mental Illness, Criminal Behaviour | Justice |
| 2021 | A Population-Based Examination of Suicide and Child Protection System Involvement | (Palmer et al., 2021) | 170 | USA  (California) | Child | Physical, Psychological, Sexual | Health |
| 2021 | Accidental injury, self-injury, and assault among children of women with schizophrenia: a population-based cohort study | (Taylor et al., 2021) | 3,769 | Canada  (Ontario) | Prenatal | Mental Illness | Health |
| 2021 | Adverse childhood experiences and asthma: trajectories in a national cohort | (Pape et al., 2021) | 466,556 | Denmark | Birth – 2 yrs | Mental Illness | Health |
| 2021 | Adverse childhood experiences and child mental health: an electronic birth cohort study | (Lowthian et al., 2021) | 191,035 | United Kingdom  (Wales) | Birth<1 yr  1<5 yrs  5<8 yrs  8<12 yrs | Mental Illness, Substance Abuse | Health |
| 2021 | Association of Child Maltreatment With Risk of Death During Childhood in South Australia | (Segal, Doidge, et al., 2021) | 608,547 | Australia  (South Australia) | Infancy – 16 yrs | Physical, Psychological, Sexual | Health |
| 2021 | Association of Youth Age at Exposure to Household Dysfunction With Outcomes in Early Adulthood | (Andersen, 2021) | 605,344 | Denmark | 0-2 yrs  3-5 yrs  6-12 yrs  13-17 yrs | Criminal Behaviour, Mental Illness, Physical, Psychological, Sexual | Health, Education |
| 2021 | Child Maltreatment and Mortality in Young Adults | (Segal, Armfield, et al., 2021) | 331,254 | Australia  (South Australia) | <16 yrs | Physical, Psychological, Sexual | Health |
| 2021 | Early childhood predictors of elementary school suspension: An Australian record linkage study | (Laurens et al., 2021) | 34,855 | Australia | Child | Mental Illness, Criminal Behaviour | Education |
| 2021 | Early school suspensions for children with adverse childhood experiences (ACEs) | (Bell et al., 2021) | 15,109 | Australia  (Western Australia) | Conception – end Grade 3 | Physical, Psychological, Sexual, Mental Illness, Criminal Behaviour | Education |
| 2021 | Educational and health outcomes of schoolchildren in local authority care in Scotland: A retrospective record linkage study | (Fleming et al., 2021) | 715,111 | United Kingdom  (Scotland) | 4-18 yrs | Physical, Psychological, Sexual | Education, Health |
| 2021 | Evaluating abusive head trauma in children <5years old: Risk factors and the importance of the social history | (Notrica et al., 2021) | 782 | USA  (Phoenix) | < 5 yrs | Domestic violence, Mental Illness, Substance Abuse, Physical, Psychological, Sexual | Health |
| 2021 | Family and Child Risk Factors for Early-Life RSV Illness | (Fitzpatrick et al., 2021) | 11,782 | Canada  (Ontario) | 2 yrs prior to birth | Criminal Behaviour, Substance Abuse, Mental Illness | Health |
| 2021 | Intergenerational transmission of child maltreatment in South Australia, 1986-2017: a retrospective cohort study | (Armfield et al., 2021) | 38,556 | Australia (South Australia) | Birth – 18 yrs | Physical, Psychological, Sexual | Welfare |
| 2021 | Opportunities for prevention: a data-linkage study to inform a public health response to youth offending in the Northern Territory, Australia | (He et al., 2021) | 10,438 | Australia  (Northern Territory) | 0-4 yrs  5-9 yrs | Physical, Psychological, Sexual | Justice |
| 2021 | Patterns of child protection service involvement by Aboriginal children associated with a higher risk of self-harm in adolescence: A retrospective population cohort study using linked administrative data | (Leckning et al., 2021) | 6,467 | Australia  (Northern Territory) | 0-4 yrs  5-11 yrs  15-18 yrs | Physical, Psychological, Sexual | Health |
| 2021 | Prenatal antidepressant exposure and child development at kindergarten age: a population-based study | (Park et al., 2021) | 94,712 | Canada  (British Columbia) | Prenatal | Mental Illness | Health, Education |
| 2021 | Risk factors for emergency room use and rehospitalization among opioid-exposed newborns in Massachusetts | (Hwang et al., 2021) | 4,524 | USA  (Massachusetts) | Prenatal | Substance Abuse, Mental Illness | Health |
| 2021 | Sobriety treatment and recovery teams for families with co-occurring substance use and child maltreatment: A propensity score-matched evaluation | (Hall et al., 2021) | 1,042 | USA  (Kentucky) | Birth – 27 dys  28 dys – 12 mos,  13 -23 mos  2-5 yrs  6-11 yrs  12-18 yrs | Mental Illness, Criminal Behaviour, Substance Abuse | Welfare |
| 2021 | The influence of child maltreatment on substance or alcohol use in 30-year-old adults: A birth cohort study. | (Kisely et al., 2021) | 2,861 | Australia  (Queensland) | Child | Physical, Psychological, Sexual | Health |
| 2021 | The roles of housing, financial, and food insecurities in understanding the relationship between childhood neglect and violence in adulthood | (Schuck & Widom, 2021) | 713 | USA  (Midwest) | <12 yrs | Physical, Psychological, Sexual | Justice |
| 2022 | "Short-stayers" in child welfare: Characteristics and system experiences | (Getz et al., 2022) | 251,112 | USA | 0-1 yrs  2-5 yrs  6-11 yrs  12-17 yrs  18+ yrs | Physical, Psychological, Sexual, Substance Abuse | Welfare |
| 2022 | Association of parental substance misuse with offspring substance misuse and criminality: a genetically informed register-based study | (Latvala et al., 2022) | 2,476,198 | Sweden | <18 yrs | Mental Illness, Substance Abuse | Health, Justice |
| 2022 | Child maltreatment and emergency department visits: a longitudinal birth cohort study from infancy to early adulthood | (Gnanamanickam et al., 2022) | 443,754 | Australia  (Adelaide) | 0-12 yrs  13-17 yrs  18+ yrs | Physical, Psychological, Sexual | Health, Welfare |
| 2022 | Child maltreatment and substance abuse: Unaccompanied children and adverse outcomes in long-term foster care in the United States | (Hasson et al., 2022) | 204 | USA | Child | Physical, Psychological, Sexual | Health |
| 2022 | Child Protection Intervention and the Sexual and Reproductive Health of Female Adolescents Ages 13 to 17 Years | (Font et al., 2022) | 9,392 | USA  (Wisconsin) | <13-18 yrs | Physical, Psychological, Sexual | Health |
| 2022 | Child protection involvement of children of mothers with intellectual disability | (Lima et al., 2022) | 1,106 | Australia (Western Australia) | Infants  1-5 yrs  6-10 yrs  11-18 yrs | Substance Abuse, Mental Illness | Welfare |
| 2022 | Childhood maltreatment and repeat offending in juvenile delinquents: A propensity score matched-control study | (Cho & Lee, 2022) | 698 | USA  (Minnesota) | <10-15 yrs | Physical, Psychological, Sexual | Justice |
| 2022 | Community Adversity and Utilization of Psychotropic Medications Among Children in Foster Care | (Bozzi et al., 2022) | 4,334 | USA | 5-18 yrs | Physical, Psychological, Sexual | Health |
| 2022 | Developmental profiles of schizotypy in the general population: A record linkage study of Australian children aged 11-12 years | (Green et al., 2022) | 22,137 | Australia  (New South Wales) | <5-6 yrs | Physical, Psychological, Sexual | Health |
| 2022 | Differences in Mortality Among Infants With Neonatal Opioid Withdrawal Syndrome | (Austin et al., 2022) | 4,480 | USA  (North Carolina) | Prenatal | Substance Use | Health |
| 2022 | Early developmental vulnerabilities following exposure to domestic violence and abuse: Findings from an Australian population cohort record linkage study | (Whitten et al., 2022) | 82,501 | Australia  (New South Wales) | Birth – 14 yrs | Criminal Behaviour | Health, Education |
| 2022 | Enrollment and outcomes of home visiting for mothers with and without a history of out-of-home care | (Nause et al., 2022) | 1,375 | USA  (Ohio) | Childhood | Physical, Psychological, Sexual | Health |
| 2022 | Epilepsy in children exposed to family and domestic violence in the first 5 years of life | (Orr, Fisher, et al., 2022) | 7,018 | Australia  (Western Australia) | <5 yrs | Domestic Violence | Health |
| 2022 | Evaluation of pushing out of children from all English state schools: Administrative data cohort study of children receiving social care and their peers | (Jay et al., 2022) | 1,081,779 | England | 8-11 yrs | Physical, Psychological, Sexual | Education |
| 2022 | Evidencing Predictors of Adolescent to Parent Violence Re-Offending Through Linkage of Police and Health Records | (Peck et al., 2022) | 755 | Australia  (New South Wales) | <13 yrs | Domestic Violence, Substance Abuse, Psychological, Physical, Sexual | Health, Justice |
| 2022 | Examining Housing Instability Among Females Who Are System-Involved: Comparing Females With and Without Histories of Commercial Sexual Exploitation | (Dierkhising et al., 2022) | 471 | USA  (California) | Child | Sexual | Justice |
| 2022 | Examining the intersection of child protection and public housing: Development, health and justice outcomes using linked administrative data | (Malvaso et al., 2022) | 67,454 | Australia  (South Australia) | <16 yrs | APhysical, Psychological, Sexual | Welfare, Health, Education, Justice |
| 2022 | Intergenerational transmission of suicide attempt in a cohort of 4.4 million children | (Ranning et al., 2022) | 4,419,651 | Denmark | Prenatal  0-1 yrs  2-5 yrs  6-12 yrs  13-17 yrs  18-24 yrs | Mental Illness, Substance Abuse | Health |
| 2022 | Investigating adjustment profiles in children exposed to intimate partner violence using a biopsychosocial resilience framework: A Canadian population-based study | (Cameranesi et al., 2022) | 3,886 | Canada  (Manitoba) | Prenatal  <10 yrs | Domestic Violence, Mental Illness | Health |
| 2022 | Maternal schizophrenia and the risk of a childhood chronic condition | (Vigod et al., 2022) | 5,066 | Canada  (Ontario) | <19 yrs | Mental Illness | Health |
| 2022 | Neglect subtypes in relation to rereport and foster care entry outcomes | (Chiang et al., 2022) | 7,313 | USA  (St Louis) | <11 yrs | Physical, Psychological, Sexual, Substance Abuse, Criminal Behaviour | Welfare |
| 2022 | Parental and community risk factors for childhood self-harm thoughts and behaviours | (O'Hare et al., 2022) | 74,479 | Australia  (New South Wales) | <14 yrs | Criminal Behaviour, Mental Illness | Health |
| 2022 | Patterns of healthcare utilization with placement changes for youth in foster care | (Beal et al., 2022) | 2,787 | USA  (Midwest) | 4-21 yrs | Physical, Psychological, Sexual | Health |
| 2022 | Reunification trajectories in Quebec: Acknowledging chronic family challenges to support stability | (Esposito et al., 2022) | 1,120 | Canada  (Quebec) | Children | Domestic Violence, Mental Illness, Substance Abuse, Criminal Behaviour | Welfare |
| 2022 | Risk of non-fatal suicide attempt in individuals with substance use disorder: the roles of aggregate genetic liability and environmental exposures in a Swedish population-based cohort | (Lannoy et al., 2022) | 228,617 | Sweden | 0-15 yrs | Substance Abuse | Health |
| 2022 | School Readiness of Children Exposed to Family and Domestic Violence | (Orr, O’Donnell, et al., 2022) | 7,016 | Australia  (Western Australia) | 2 yrs prior to birth | Domestic Violence | Education, Health |
| 2022 | The Association of Race, Ethnicity, and Poverty With Child Maltreatment Reporting | (Rebbe et al., 2022) | 3,907 | USA  (Washington State) | <3 yrs | Physical, Psychological, Sexual | Welfare |
| 2022 | The extent of violence inflicted on adolescent Aboriginal girls in the Northern Territory | (Moore et al., 2022) | 13,531 | Australia  (Northern Territory) | <12 yrs | Physical, Psychological, Sexual | Justice |
| 2023 | A Longitudinal Analysis of Concerning Psychotropic Medication Regimens Among Adolescents in Foster Care | (L. Palmer et al., 2023) | 2,998 | USA  (Wisconsin) | 10-13 yrs | Physical, Psychological, Sexual | Health |
| 2023 | Academic performance in adolescent offspring of mothers with prenatal and perinatal psychiatric hospitalizations: A register-based, data linkage, cohort study | (Ayano et al., 2023) | 168,528 | Australia  (New South Wales) | Before, during and one year after pregnancy | Mental Illness | Education |
| 2023 | Advancing Research: An Examination of Differences in Characteristics of Sexual and Non-Sexual Offense Recidivism Using a 10-Year Follow-Up | (Zgoba et al., 2023) | 626 | USA | Childhood | Physical, Sexual | Justice |
| 2023 | Child protection system involvement in children of incarcerated mothers: A linked data study | (Segal et al., 2023) | 6,680 | Australia  (Western Australia) | <18 yrs | Criminal Behaviour | Welfare |
| 2023 | Conditions of Birth and Early Childhood Developmental Risk for Mental Disorders | (Harris et al., 2023) | 66,646 | Australia  (New South Wales) | Prenatal | Mental Illness | Health, Education |
| 2023 | Cumulative Environmental Risk in Early Life: Associations with Schizotypy in Childhood | (O'Hare, Watkeys, et al., 2023) | 20,599 | Australia  (New South Wales) | Prenatal – 11 yrs | Physical, Psychological, Sexual, Criminal Behaviour | Health |
| 2023 | Early childhood adversity and body mass index in childhood and adolescence: linking registry data on adversities with school health records of 53,401 children from Copenhagen | (Elsenburg et al., 2023) | 53,401 | Denmark  (Copenhagen) | 0-5 yrs | Mental Illness, Substance Abuse | Health |
| 2023 | Early childhood developmental vulnerability associated with parental mental disorder comorbidity | (Watkeys et al., 2023) | 66,154 | Australia  (New South Wales) | <5 yrs | Mental Illness | Health, Education |
| 2023 | Early predictors for maltreatment-related injuries in infancy and long-term mortality: a population-based study | (Chen et al., 2023) | 6,297 | Taiwan | 0-3 months  4-7 months  8-11 months | Physical, Psychological, Sexual, Mental Illness | Health |
| 2023 | Examining the relationship between maternal mental health-related hospital admissions and childhood developmental vulnerability at school entry in Canada and Australia | (Bell, Glauert, et al., 2023) | 89,314 | Canada  (Manitoba)  and  Australia  (Western Australia) | Pregnancy,  birth to 1 yr,  1-2 yrs,  3-4 yrs,  4 yrs to school entry | Mental Illness | Health, Education |
| 2023 | Exposure to family and domestic violence is associated with lower attendance and higher suspension in school children | (C. Orr et al., 2023) | 23,743 | Australia  (Western Australia) | Child | Domestic Violence | Education |
| 2023 | Factors associated with low school readiness, a linked health and education data study in Wales, UK | (Bandyopadhyay, Marchant, et al., 2023) | 14,733 | Wales | Prenatal | Mental Illness, Substance Abuse, Domestic Violence | Education |
| 2023 | Factors Associated With Permanency for Children in Out-Of-Home Placement: A Survival Analysis | (Ahn et al., 2023) | 2,379 | USA  (Mid Atlantic State) | 0-3 yrs  4-9 yrs  10-16 yrs | Physical, Psychological, Sexual | Welfare |
| 2023 | Father-Mother Co-Involvement in Child Maltreatment: Associations of Prior Perpetration, Parental Substance Use, Parental Medical Conditions, Inadequate Housing, and Intimate Partner Violence with Different Maltreatment Types | (Lee et al., 2023) | 26,195 | USA | Child | Physical, Psychological, Sexual, Domestic Violence, Mental Illness | Welfare |
| 2023 | Homotypical and Heterotypical Intergenerational Continuity of Child Maltreatment: Evidence from a Cohort of Families Involved with Child Protection Services | (Langevin et al., 2023) | 5,861 | Canada (Quebec) | 0-17 yrs | Physical, Psychological, Sexual, Domestic Violence | Welfare |
| 2023 | Hospital inpatient admissions of children of mothers with severe mental illness: A Western Australian cohort study | (Pierce et al., 2023) | 467,945 | Australia  (Western Australia) | <5 yrs | Mental Illness | Health |
| 2023 | Identifying Risk Factors and Advancing Services for Violently Injured Low-Income Black Youth | (Voith et al., 2023) | 452 | USA  (Cleveland, Ohio) | Child | Physical, Psychological, Sexual | Health |
| 2023 | Impact of socio-economic conditions and perinatal factors on risk of becoming a child looked after: a whole population cohort study using routinely collected data in Wales | (Melis et al., 2023) | 396,610 | United Kingdom  (Wales) | Perinatal period (pregnancy to 1 yr) | Mental Illness, Substance Abuse | Welfare |
| 2023 | Maternal incarceration increases the risk of self-harm but not suicide: a matched cohort study | (Cumming et al., 2023) | 7,674 | Australia  (Western Australia) | Pregnancy – 18 yrs | Physical, Psychological, Sexual, Criminal Behaviour, Mental Illness | Health |
| 2023 | Mental health-related hospitalisations associated with patterns of child protection and youth justice involvement during adolescence: A retrospective cohort study using linked administrative data from the Northern Territory of Australia | (Leckning et al., 2023) | 14,972 | Australia  (Northern Territory) | 10-18 years | Physical, Psychological, Sexual | Health |
| 2023 | Neonatal abstinence syndrome and other neonatal outcomes for the infants of women experiencing incarceration: A retrospective cohort study | (Bell, Kelty, et al., 2023) | 9,741 | Australia  (Western Australia) | 1 year prior to pregnancy, during pregnancy, 1 year post pregnancy | Criminal Behaviour | Health |
| 2023 | Numeracy and literacy attainment of children exposed to maternal incarceration and other adversities: A linked data study | (Bell, Segal, et al., 2023) | 13,398 | Australia  (Western Australia) | Pregnancy – 18 yrs | Criminal Behaviour, Mental Illness. Physical, Psychological, Sexual | Education |
| 2023 | Out-of-home care characteristics associated with childhood educational underachievement, mental disorder, and police contacts in an Australian population sample | (O'Hare, Tzoumakis, et al., 2023) | 2,082 | Australia  (New South Wales) | 0-13 yrs | Physical, Psychological, Sexual | Education, Health, Justice |
| 2023 | Patterns of substance use among adolescents in and out of foster care: An analysis of linked health and child welfare administrative data | (Beal et al., 2023) | 2,787 | USA | 10-20 yrs | Physical, Psychological, Sexual, Mental Illness | Health |
| 2023 | Perinatal outcomes of Aboriginal women with mental health disorders | (Adane et al., 2023) | 38,592 | Australia  (Western Australia) | Before or during pregnancy | Substance Abuse, Mental Illness | Health |
| 2023 | Poor Neonatal Adaptation After Antidepressant Exposure During the Third Trimester in a Geographically Defined Cohort | (Brumbaugh et al., 2023) | 1,262 | USA  (Minnesota) | Prenatal | Mental Illness, Substance Abuse | Health |
| 2023 | Predicting childhood and adolescent attention-deficit/hyperactivity disorder onset: a nationwide deep learning approach | (Garcia-Argibay et al., 2023) | 238,696 | Sweden | 0-19 yrs | Criminal Behaviour, Mental Illness | Health |
| 2023 | Process mining the trajectories for adolescent-to-mother violence from longitudinal police and health service data | (Peck et al., 2023) | 775 | Australia  (New South Wales) | 0-19 yrs | Substance Abuse, Mental Illness, Physical, Psychological, Sexual, Domestic Violence | Justice |
| 2023 | Relationships between parental mental illness and/or offending and offspring contact with the police in childhood: Findings from a longitudinal record-linkage study | (Athanassiou et al., 2023) | 72,771 | Australia  (New South Wales) | <5 yrs | Mental Illness, Domestic Violence | Justice |
| 2023 | Reprint of: Homelessness and child protection involvement: Temporal links and risks to student attendance and school mobility | (A. R. Palmer et al., 2023) | 3,278 | USA (Minnesota) | 4-15 yrs | Physical, Psychological, Sexual | Education |
| 2023 | Reproductive and sexual health of Australian adolescents exposed to family and domestic violence | (Carol Orr et al., 2023) | 58,352 | Australia  (Western Australia) | 0-18 yrs | Criminal Behaviour, Domestic Violence | Health |
| 2023 | Risk of school exclusion among adolescents receiving social care or special educational needs services: A whole-population administrative data cohort study | (Jay et al., 2023) | 1,031,500 | England | (School years) 4-6  7-9 | Physical, Psychological, Sexual | Education |
| 2023 | Self-reported mental health of children known to child protection services: an Australian population-based record linkage study | (O'Hare, Hussain, et al., 2023) | 26,960 | Australia  (New South Wales) | <10 yrs | Physical, Psychological, Sexual | Health |
| 2023 | Three-Year Custody Outcomes Among Infants Investigated by Child Protection Systems for Prenatal Substance Exposure in California | (Reddy et al., 2023) | 22,855 | USA  (California) | Prenatal | Substance Abuse | Welfare |
| 2023 | Weighting of risk factors for low birth weight: A linked routine data cohort study in Wales | (Bandyopadhyay, Jones, et al., 2023) | 693,377 | United Kingdom  (Wales) | Prenatal | Mental Illness, Substance Abuse | Health |
| 2024 | A Longitudinal Study of Health Care Utilization Among Infants Investigated for Maltreatment | (Vega et al., 2024) | 3,692 | USA  (California) | <1 yr | Physical, Psychological, Sexual | Health |
| 2024 | Association of maternal risk factors with infant maltreatment: an administrative data cohort study | (Smith et al., 2024) | 434 | Canada  (Ontario) | Infant – 1 yr | Mental Illness, Substance Abuse, Domestic Violence | Welfare, Health |
| 2024 | Childhood contact with social services and risk of suicide or sudden death in young adulthood: identifying hidden risk in a population-wide cohort study | (McKenna et al., 2024) | 437,008 | Northern Ireland | <18 yrs | Physical, Psychological, Sexual | Health |
| 2024 | Infants entering out-of-home care: Health, developmental needs and service provision | (Lima et al., 2024) | 474 | Australia  (New South Wales) | <1 yr | Physical, Psychological, Sexual, Substance Abuse | Health |
